# Atypical response to bacterial coinfection and persistent neutrophilic bronchoalveolar inflammation distinguish critical COVID-19 from influenza

**DOI:** 10.1172/jci.insight.155055

**Published:** 2022-01-11

**Authors:** Seppe Cambier, Mieke Metzemaekers, Ana Carolina de Carvalho, Amber Nooyens, Cato Jacobs, Lore Vanderbeke, Bert Malengier-Devlies, Mieke Gouwy, Elisabeth Heylen, Philippe Meersseman, Greet Hermans, Els Wauters, Alexander Wilmer, the CONTAGIOUS Consortium, Dominique Schols, Patrick Matthys, Ghislain Opdenakker, Rafael Elias Marques, Joost Wauters, Jennifer Vandooren, Paul Proost

**Affiliations:** 1 Laboratory of Molecular Immunology, Department of Microbiology, Immunology and Transplantation, Rega Institute, KU Leuven, Leuven, Belgium.; 2 Brazilian Biosciences National Laboratory (LNBio), Brazilian Center for Research in Energy and Materials (CNPEM), Campinas, Brazil.; 3 Department of Genetics, Microbiology and Immunology, Institute of Biology, University of Campinas (UNICAMP), Campinas, Brazil.; 4 Laboratory for Clinical Infectious and Inflammatory Disorders and; 5 Laboratory of Clinical Bacteriology and Mycology, Department of Microbiology, Immunology and Transplantation, KU Leuven, Leuven, Belgium.; 6 Laboratory of Immunobiology and; 7 Laboratory of Virology and Chemotherapy, Department of Microbiology, Immunology and Transplantation, Rega Institute, KU Leuven, Leuven, Belgium.; 8 Laboratory of Intensive Care Medicine, Department of Cellular and Molecular Medicine, and; 9 Laboratory of Respiratory Diseases and Thoracic Surgery (BREATHE), Department of Chronic Diseases and Metabolism, KU Leuven, Leuven, Belgium.; 10 Department of Pneumology, University Hospitals Leuven, Leuven, Belgium.; 11 The CONTAGIOUS Consortium is detailed in Supplemental Acknowledgments.

**Keywords:** COVID-19, Immunology, Cytokines, Influenza, Neutrophils

## Abstract

Neutrophils are recognized as important circulating effector cells in the pathophysiology of severe coronavirus disease 2019 (COVID-19). However, their role within the inflamed lungs is incompletely understood. Here, we collected bronchoalveolar lavage (BAL) fluids and parallel blood samples of critically ill COVID-19 patients requiring invasive mechanical ventilation and compared BAL fluid parameters with those of mechanically ventilated patients with influenza, as a non–COVID-19 viral pneumonia cohort. Compared with those of patients with influenza, BAL fluids of patients with COVID-19 contained increased numbers of hyperactivated degranulating neutrophils and elevated concentrations of the cytokines IL-1**β**, IL-1RA, IL-17A, TNF-**α**, and G-CSF; the chemokines CCL7, CXCL1, CXCL8, CXCL11, and CXCL12**α**; and the protease inhibitors elafin, secretory leukocyte protease inhibitor, and tissue inhibitor of metalloproteinases 1. In contrast, **α**-1 antitrypsin levels and net proteolytic activity were comparable in COVID-19 and influenza BAL fluids. During antibiotic treatment for bacterial coinfections, increased BAL fluid levels of several activating and chemotactic factors for monocytes, lymphocytes, and NK cells were detected in patients with COVID-19 whereas concentrations tended to decrease in patients with influenza, highlighting the persistent immunological response to coinfections in COVID-19. Finally, the high proteolytic activity in COVID-19 lungs suggests considering protease inhibitors as a treatment option.

## Graphical abstract

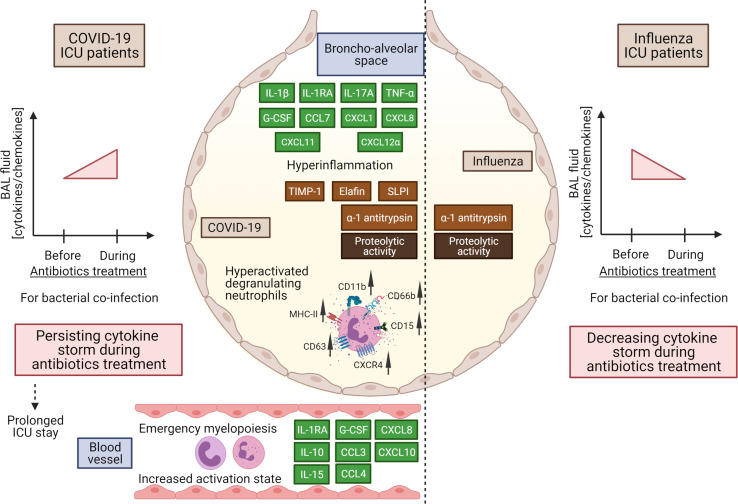

## Introduction

Severe acute respiratory syndrome coronavirus 2 (SARS-CoV-2) infection and the resulting coronavirus disease 2019 (COVID-19) continue to pose a major threat to global health and the global economy, with more than 267 million confirmed cases and more than 5.27 million deaths as of December 2021 (1). COVID-19 is heterogeneous in its severity, with most patients being asymptomatic or facing mild symptoms. However, up to 20% of patients develop severe acute respiratory distress syndrome (ARDS), thus requiring intensive care (2). The systemic hyperinflammatory response in severe COVID-19 is associated with dysregulation of the immune system and is characterized by an atypical cytokine storm, lymphopenia, and increased neutrophil counts in blood (3–5). Neutrophils, as the most abundant circulating leukocytes in humans, are among the first responders to infection, exploiting a multitude of oxidative and nonoxidative effector mechanisms (6, 7). In the blood of patients with severe COVID-19, the presence of immature neutrophils has been evidenced, indicating a situation of emergency myelopoiesis (8–10). Besides, a state of increased neutrophil activation in the circulation, with elevated levels of neutrophil-mobilizing/activating factors, neutrophil-derived proteases, and neutrophil extracellular traps (NETs) associated with immunothrombosis, is observed in critically ill patients (8, 11–14). Moreover, myeloid-derived suppressor cell–like neutrophils with an immunosuppressive effect on T cells are seen (9, 15, 16). Thus, growing consensus exists that neutrophils are key effector cells in severe COVID-19. Therefore, a better understanding of the role of neutrophils that have infiltrated the lungs is required. Within the bronchoalveolar space, increased neutrophil counts with a heterogeneous phenotype are seen, but most information is available from single-cell transcriptomics studies whereas information on protein levels is limited (17–19). Moreover, the effect on the inflammatory response of bacterial or fungal coinfection(s) in mechanically ventilated COVID-19 patients requires further investigation. Indeed, ventilator-associated lower respiratory tract infections are significantly more prevalent in COVID-19 compared with influenza patients or ventilated patients without viral infections (20) and are associated with a longer duration of ventilation, hospitalization in intensive care units (ICUs), and mortality (21, 22).

Here, we collected blood and bronchoalveolar lavage (BAL) fluid samples from critically ill COVID-19 patients hospitalized in ICUs and requiring invasive mechanical ventilation or extracorporeal membrane oxygenation (ECMO). The aim was to phenotypically characterize neutrophils, determine cytokine/chemokine levels, define the protease/protease inhibitor balances within the lungs, and study the effect of coinfections in this context. The obtained results were compared with those of blood samples from healthy volunteers and with BAL samples from critically ill influenza patients in the ICU, as a non–COVID-19 viral pneumonia control group.

## Results

### Patient cohort.

**Figure 1:**
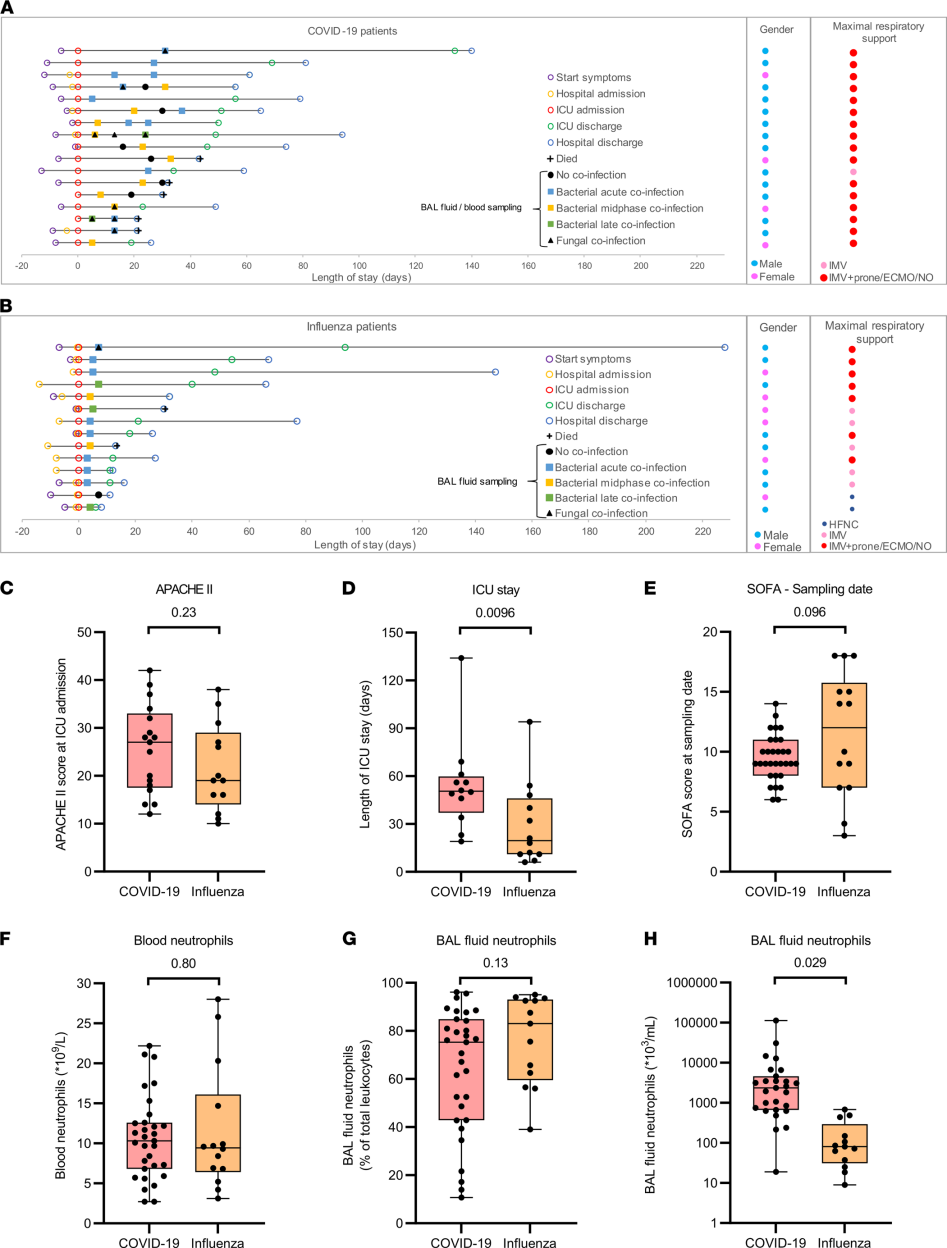
COVID-19 and influenza patient characteristics. (**A** and **B**) Clinical course timeline of the (**A**) COVID-19 (*n* = 17) and (**B**) influenza (*n* = 14) ICU patients. Patients are ranked based on the length of ICU stay with time point 0 representing ICU admission. The coinfection status at the moment of BAL/blood sampling is indicated. Samples were categorized based on the absence of a coinfection or the acute phase (clinical/biochemical worsening and antibiotics not yet or recently started), midphase (signs of improvement with ongoing antibiotic therapy), or late phase (final days of antibiotic therapy nearing complete remission) of a bacterial coinfection based on the timing of the BAL sample analyzed relative to the coinfection time course. A fungal coinfection was diagnosed based on radiological abnormalities in combination with clinical signs and mycological evidence (positive galactomannan in BAL and/or serum and/or presence of *Aspergillus fumigatus* in BAL culture). Next to the timeline, sex and maximal respiratory support during hospital stay are shown. (**C**) Acute physiology and chronic health evaluation II (APACHE II) score at ICU admission and (**D**) length of ICU stay of all patients included in the study (patients who died are excluded). (**E**) Sequential Organ Failure Assessment (SOFA) score at the moment BAL fluid and blood samples were collected from COVID-19 (*n* = 31) and influenza patients (*n* = 14). (**F**–**H**) Blood and BAL fluid neutrophil counts within the samples collected. Data are shown as box-and-whisker plots (box: median with interquartile range, whiskers: full data distribution), with each dot representing an individual patient (sample), and statistically analyzed by a Mann-Whitney *U* test or a linear mixed model with correction for multiple samples per patient using a random intercept model, where appropriate. *P* values are shown above brackets (**C**–**H**). ECMO, extracorporeal membrane oxygenation; HFNC, high-flow nasal cannula; IMV, invasive mechanical ventilation.

Seventeen COVID-19 and 14 critically ill influenza patients were recruited at the ICU of the University Hospital Leuven (Figure 1, A and B; Table 1; and Table 2). Patients with COVID-19 had a comparable APACHE II score at ICU admission but stayed for a significantly longer period in the ICU compared with patients with influenza (Figure 1, C and D). All patients with COVID-19 and the vast majority of patients with influenza had invasive mechanical ventilation as the minimum level of respiratory support, with no significant differences in the SOFA scores of the COVID-19 compared with the influenza patients at the moment of BAL and blood sampling (Figure 1E). Blood neutrophil counts and the proportion of neutrophils (as a percentage of total leukocytes) in the BAL fluid were not significantly different between patients with COVID-19 and with influenza (Figure 1, F and G). However, the absolute neutrophil count in the BAL fluid was significantly increased in COVID-19 compared with influenza patients (Figure 1H). Table 1 and Table 2 contain detailed characteristics of patients included in this study.

### Hyperactivated neutrophils acquiring a novel repertoire of surface proteins in BAL fluid from patients with COVID-19.

**Figure 2:**
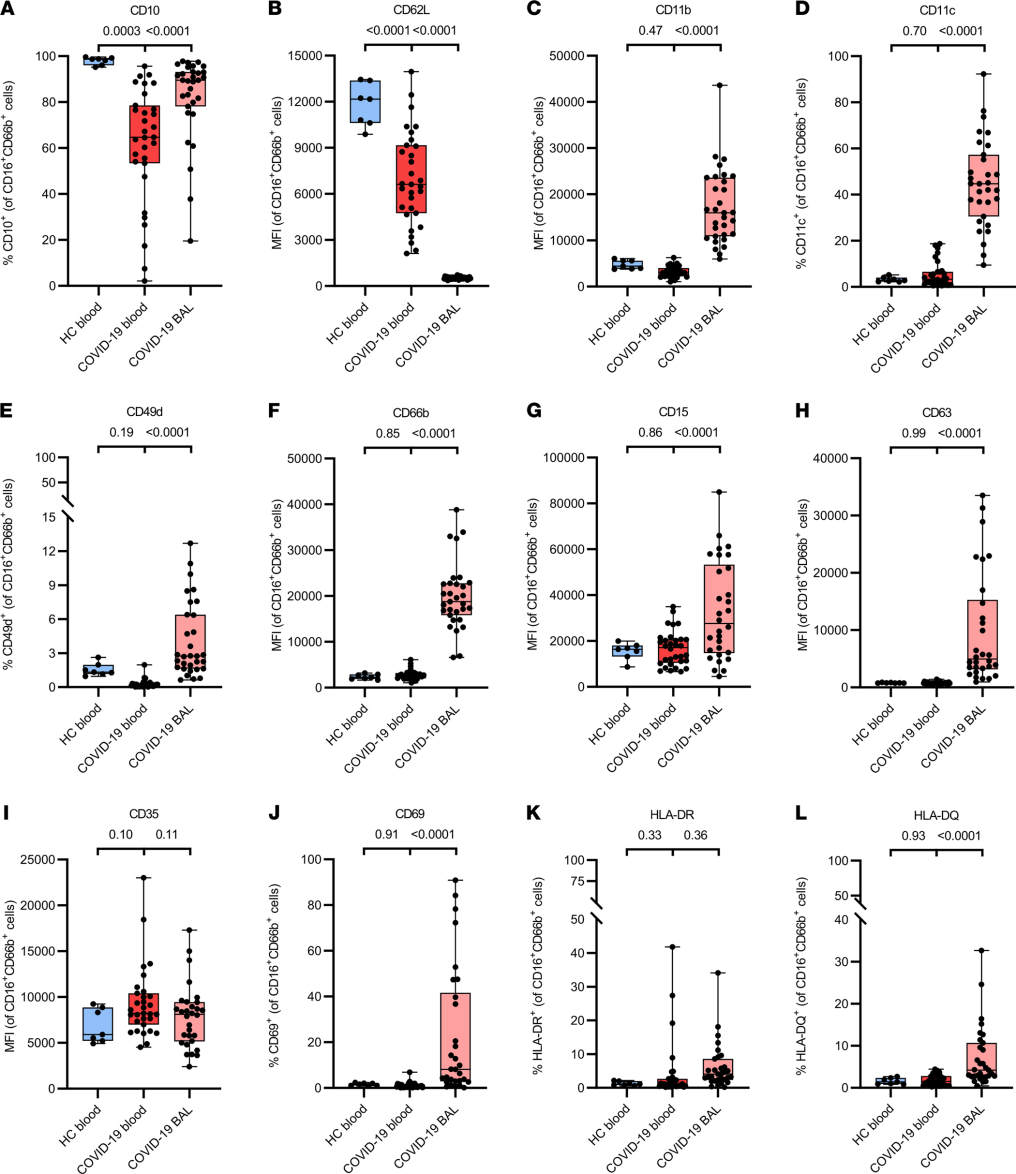
Phenotypical characterization of adhesion molecules and activation/maturation markers on BAL fluid and peripheral blood neutrophils from patients with severe COVID-19. Flow cytometry was used to evaluate the surface expression of (**A**) CD10, (**B**) CD62L, (**C**) CD11b, (**D**) CD11c, (**E**) CD49d, (**F**) CD66b, (**G**) CD15, (**H**) CD63, (**I**) CD35, (**J**) CD69, (**K**) HLA-DR, and (**L**) HLA-DQ on neutrophils (gated as CD16^+^CD66b^+^ cells) from paired blood and BAL fluid samples from COVID-19 patients (*n* = 31) and blood samples from healthy controls (HC) (*n* = 7). Results represent percentages of positive neutrophils or median fluorescence intensity (MFI). Data are shown as box-and-whisker plots (box: median with interquartile range, whiskers: full data distribution), with each dot representing an individual patient sample and statistically analyzed by a linear mixed model with correction for multiple samples per patient using a random intercept model. *P* values are shown above brackets.

**Figure 3:**
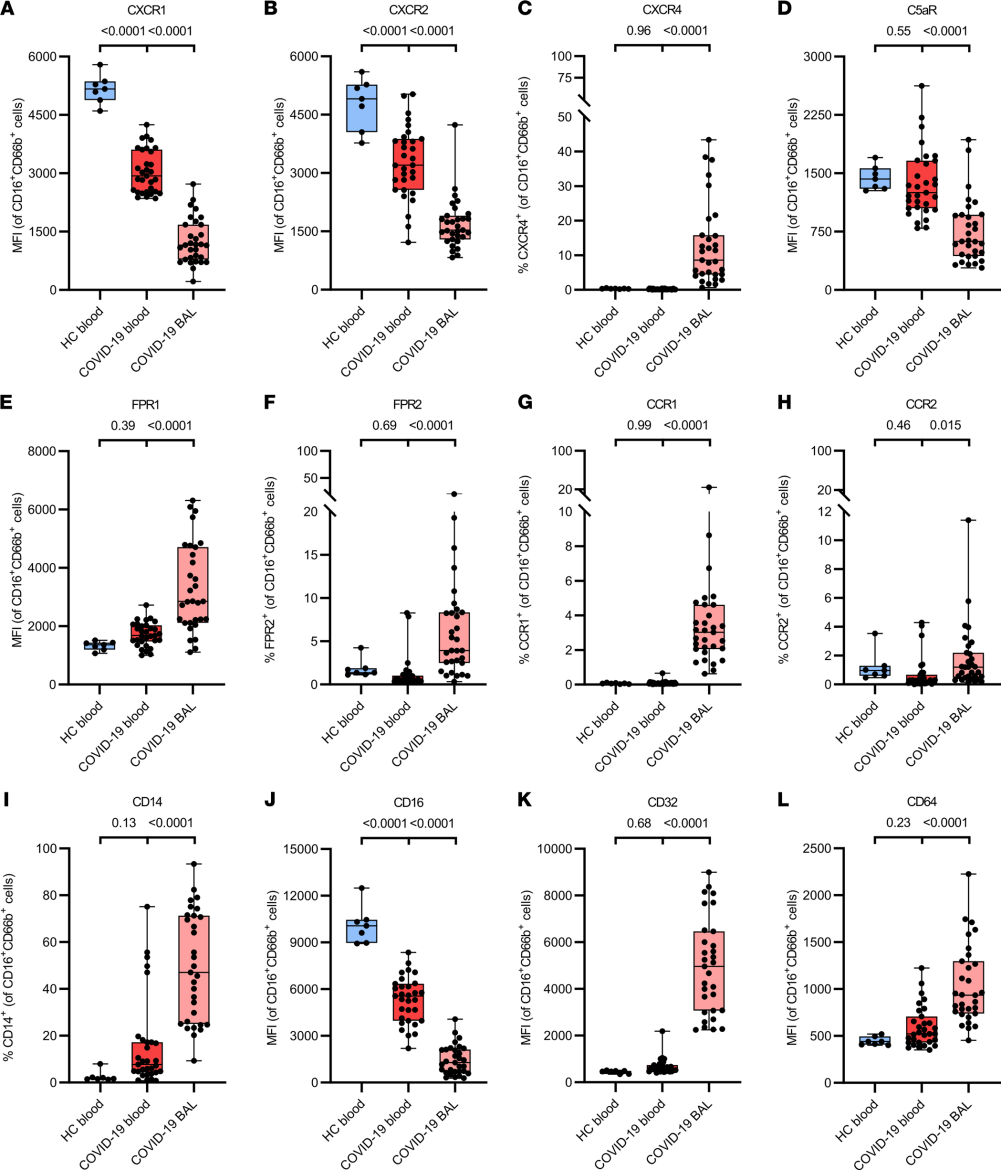
Phenotypical characterization of chemoattractant and Fcγ receptors on BAL fluid and peripheral blood neutrophils from patients with severe COVID-19. Flow cytometry was used to evaluate the surface expression of (**A**) CXCR1, (**B**) CXCR2, (**C**) CXCR4, (**D**) C5aR, (**E**) FPR1, (**F**) FPR2, (**G**) CCR1, (**H**) CCR2, (**I**) CD14, (**J**) CD16, (**K**) CD32, and (**L**) CD64 on neutrophils (gated as CD16^+^CD66b^+^ cells) from paired blood and BAL fluid samples from COVID-19 patients (*n* = 31) and blood samples from healthy controls (HC) (*n* = 7). Results represent percentages of positive neutrophils or MFI. Data are shown as box-and-whisker plots (box: median with interquartile range, whiskers: full data distribution), with each dot representing an individual patient sample, and statistically analyzed by a linear mixed model with correction for multiple samples per patient using a random intercept model. *P* values are shown above brackets.

BAL and peripheral blood neutrophils were phenotypically characterized with a focus on the expression of adhesion molecules, activation/maturation markers, Fcγ receptors, and chemoattractant receptors using multicolor flow cytometry (Figure 2 and Figure 3 and Supplemental Figure 1; supplemental material available online with this article; https://doi.org/10.1172/jci.insight.155055DS1). We confirmed the presence of immature (CD10^–^) neutrophils in the circulation of patients with COVID-19 (Figure 2A), indicating emergency myelopoiesis, as we have demonstrated before (8). BAL fluids contained significantly more mature neutrophils (>90% CD10^+^ neutrophils) in comparison with parallel blood samples. Previously, an increased neutrophil activation state was seen in the blood of COVID-19 ICU patients (8). In comparison with blood neutrophils of healthy controls, we confirmed this increased activation state in critically ill COVID-19 patients, as shown by, for example, significantly decreased expression of L-selectin (CD62L) (Figure 2B and Supplemental Figure 1A). However, BAL fluid neutrophils showed significantly more pronounced signs of activation than neutrophils in the circulation. They almost completely lacked L-selectin expression and were characterized by increased levels of the integrins α_M_ (CD11b) and α_X_ (CD11c) in comparison with matched blood neutrophils (Figure 2, B–D, and Supplemental Figure 1A). Also, a minor but significant percentage of the BAL fluid neutrophils had upregulated the α_4_ integrin CD49d (Figure 2E), which plays a role in neutrophil recruitment during bacterial lung infection in mice (23) and is upregulated by aged neutrophils (24). Moreover, in comparison with blood neutrophils, BAL neutrophils had increased expression of CD66b, Sialyl-Lewis^X^ (i.e., the selectin ligand CD15), and the tetraspanin CD63 (Figure 2, F–H, and Supplemental Figure 1B), markers that can be upregulated rapidly on the neutrophil membrane by means of degranulation (25). For complement receptor 1 (CD35), no significant differences were seen between the study groups (Figure 2I). The activation marker CD69, which is absent on quiescent neutrophils, was also detected on a significantly increased proportion of the BAL neutrophils (Figure 2J). Finally, moderate expression of the antigen-presenting MHC class II molecules HLA-DR and HLA-DQ was detected on BAL fluid neutrophils (Figure 2, K and L). The latter indicates that some of the BAL fluid neutrophils might possibly acquire antigen-presenting capacities.

Although most blood neutrophils stained positive for CXCR1 and CXCR2, the relative expression levels of these chemoattractant receptors were significantly lower on blood neutrophils from patients with COVID-19 compared with blood neutrophils from healthy controls (Figure 3, A and B; and Supplemental Figure 1, C and D). BAL fluid neutrophils displayed even lower levels of CXCR1 and CXCR2 as compared with blood cells (Figure 3, A and B), and a significant proportion of the BAL neutrophils completely lacked CXCR1 and CXCR2 (Supplemental Figure 1, C and D). In addition, some neutrophils in the BAL fluid had upregulated CXCR4, a chemokine receptor characteristic for immature or aged neutrophils (26, 27), with some patients having up to 40% CXCR4^+^ neutrophils (Figure 3C). To discriminate between these 2 subsets, we defined aged neutrophils as CXCR4^+^CD49d^+^CD10^+^ and immature neutrophils as CXCR4^+^CD49d^–^CD10^–^ and found both subsets present in the BAL samples (Supplemental Figure 1E). Among the other prototypical chemoattractant receptors present on neutrophils, the expression of complement receptor C5aR was significantly decreased (Figure 3D and Supplemental Figure 1F) whereas the formyl peptide receptors FPR1 and FPR2 were significantly increased on COVID-19 BAL fluid neutrophils (Figure 3, E and F, and Supplemental Figure 1G), in comparison with blood neutrophils. A small population of BAL fluid neutrophils (0%–20%) also expressed CCR1 or CCR2, 2 chemokine receptors that are not typically expressed on neutrophils (Figure 3, G and H). Besides, a significantly upregulated expression of CD14 (the coreceptor for lipopolysaccharide binding to Toll-like receptor 4) was seen (Figure 3I). Finally, expression of the low-affinity Fcγ receptor III (CD16) was significantly decreased whereas expression levels of Fcγ receptor II (CD32) and Fcγ receptor I (CD64) were significantly increased on neutrophils in BAL fluid compared with blood neutrophils of patients with COVID-19 (Figure 3, J–L; and Supplemental Figure 1, H and I). No significant differences were detected in the expression of IL-1 receptor 2 (IL1-R2) and the chemoattractant receptor leukotriene B_4_ receptor type 1 (Supplemental Figure 1, J and K), and no expression of IL-1R1, intercellular adhesion molecule 1, or CXCR3 was detected. In conclusion, we show with multiple parameters that neutrophils from critically ill COVID-19 patients are partially immature, activated cells in the circulation, whereas those that have migrated to the lungs are mostly mature and hyperactivated and acquire a novel repertoire of surface proteins.

### Elevated cytokine and chemokine levels in BAL fluid from COVID-19 compared with influenza patients.

**Figure 4:**
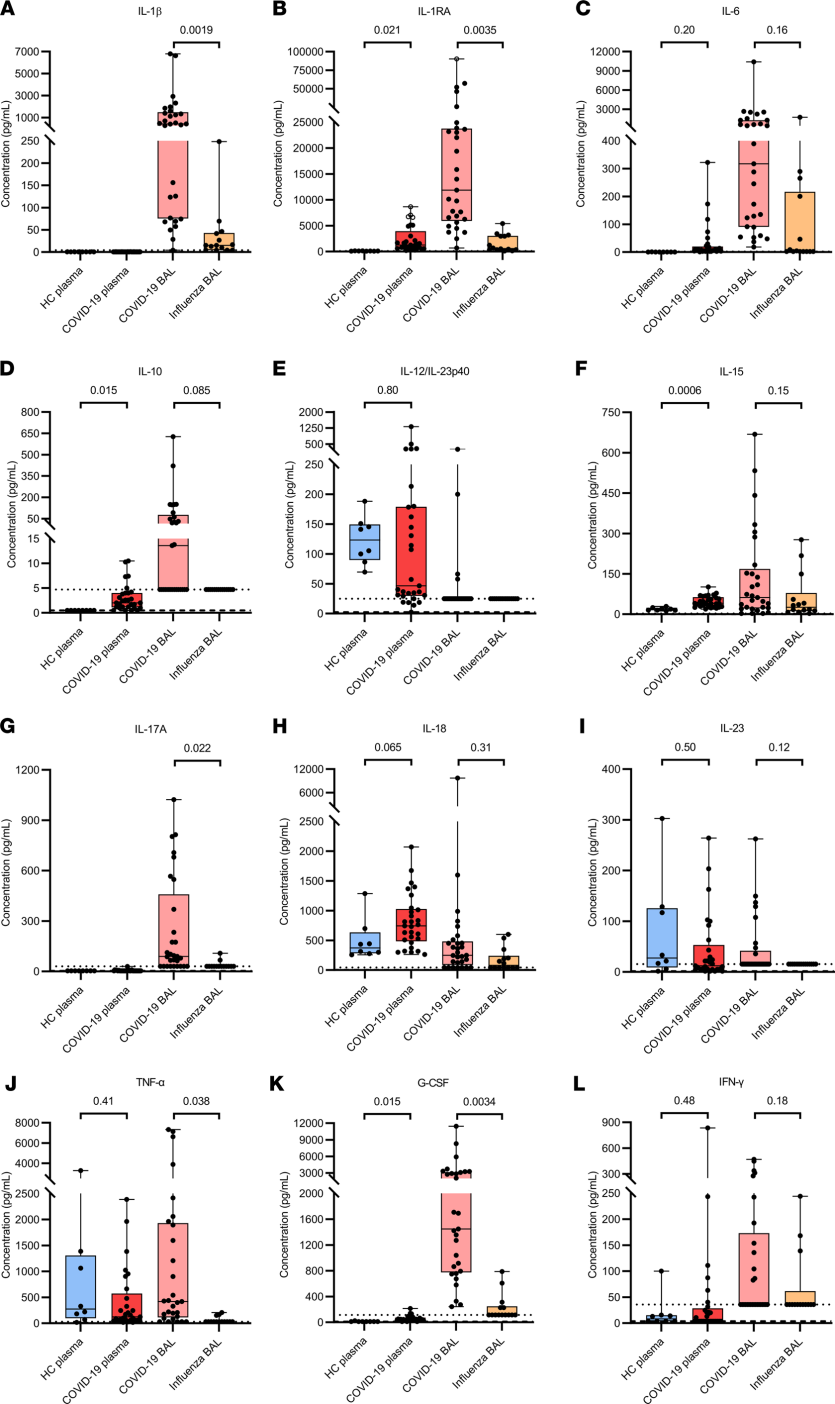
Quantification of cytokines in plasma and BAL fluid from patients with severe COVID-19 or influenza. Multiplex technology was used to determine concentrations of (**A**) IL-1β, (**B**) IL-1 receptor antagonist (IL-1RA), (**C**) IL-6, (**D**) IL-10, (**E**) IL-12/IL-23p40, (**F**) IL-15, (**G**) IL-17A, (**H**) IL-18, (**I**) IL-23, (**J**) TNF-α, (**K**) G-CSF, and (**L**) IFN-γ in plasma and BAL fluid samples from COVID-19 patients (*n* = 29), plasma samples from healthy controls (HC) (*n* = 8), and BAL fluid samples from influenza patients (*n* = 14). Data are shown as box-and-whisker plots (box: median with interquartile range, whiskers: full data distribution), with each dot representing an individual patient sample. The dashed lines indicate the lower detection limits (BAL samples were diluted 1/10). Unfilled symbols indicate values above the upper detection limit. Data were statistically analyzed by a linear mixed model with correction for multiple samples per patient using a random intercept model. *P* values are shown above brackets.

**Figure 5:**
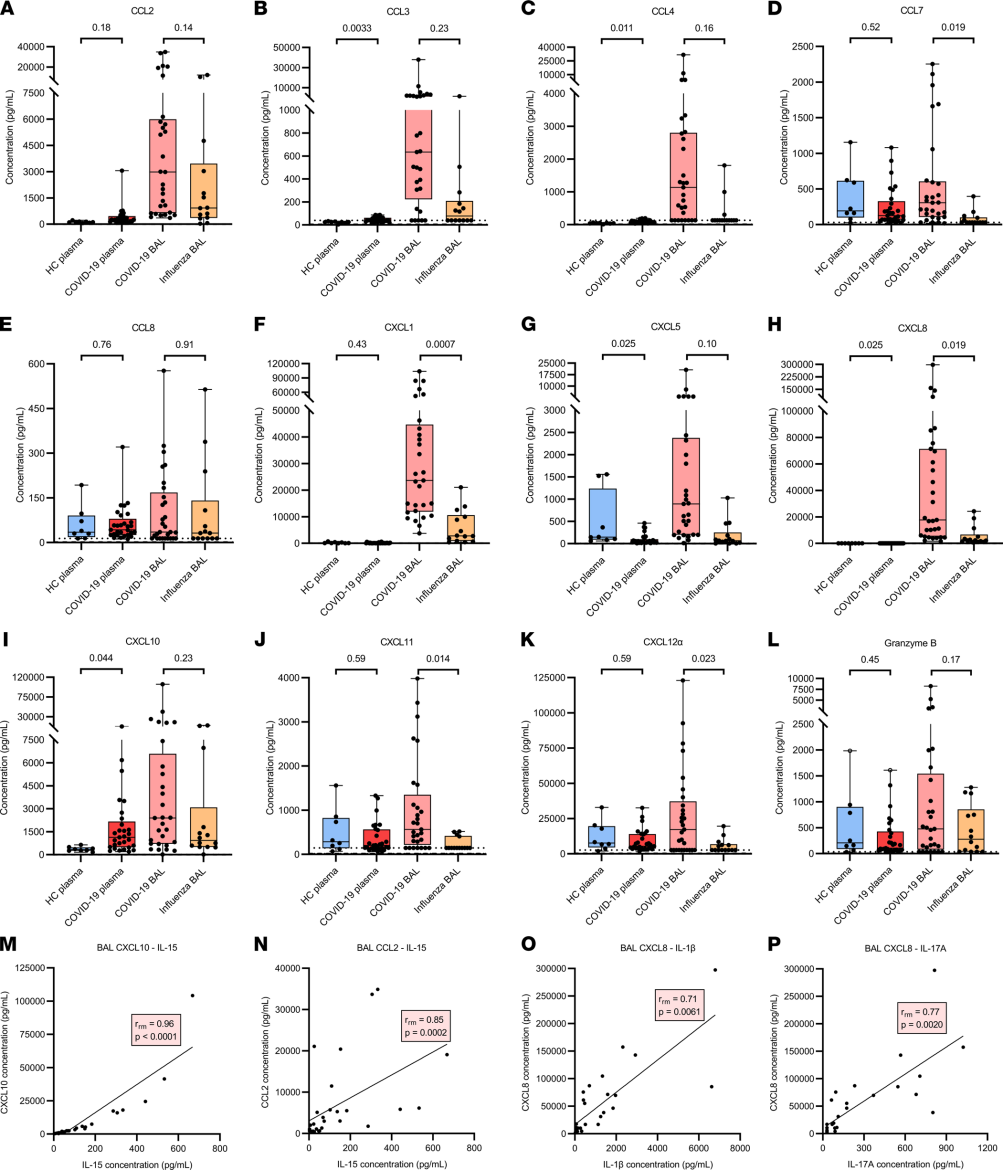
Quantification of biomarkers in plasma and BAL fluid from patients with severe COVID-19 or influenza. Multiplex and ELISA technology were used to determine concentrations of (**A**) CCL2, (**B**) CCL3, (**C**) CCL4, (**D**) CCL7, (**E**) CCL8, (**F**) CXCL1, (**G**) CXCL5, (**H**) CXCL8, (**I**) CXCL10, (**J**) CXCL11, (**K**) CXCL12α, and (**L**) granzyme B in plasma and BAL fluid samples from COVID-19 patients (*n* = 29), plasma samples from healthy controls (HC) (*n* = 8), and BAL fluid samples from influenza patients (*n* = 14). (**M**–**P**) Correlation between cytokine and chemokine levels measured in BAL fluid of COVID-19 patients. Data are shown as box-and-whisker plots (box: median with interquartile range, whiskers: full data distribution), with each dot representing an individual patient sample. The dashed lines indicate the lower detection limits (BAL samples were diluted 1/10). Data were statistically analyzed by a linear mixed model with correction for multiple samples per patient using a random intercept model. Correlation analysis was performed by calculating a repeated measures correlation coefficient and plotted utilizing a simple linear regression line. *P* values are shown above brackets.

To determine the bronchoalveolar inflammation at the protein level, cytokine and chemokine levels were quantified in BAL fluids from patients with COVID-19 and with influenza and in plasma from patients with COVID-19 and healthy controls by multiplex assays (Figure 4 and Figure 5). Plasma levels of IL-1RA, IL-10, IL-15, and G-CSF and of the chemokines CCL3, CCL4, CXCL8, and CXCL10 were significantly increased in patients with COVID-19 compared with those of healthy controls. No significant differences between patients with COVID-19 and healthy donors were detected for IFN-γ, TNF-α, granzyme B, IL-6, IL-12/IL-23p40, IL-18, IL-23, CCL2, CCL7, CCL8, CXCL1, CXCL11, and CXCL12α. Circulating GM-CSF, IL-1β, IL-4, IL-5, IL-12p70, and IL-17A concentrations were below the detection limit for most donors.

In the BAL fluid of patients with COVID-19, significantly increased and extremely high levels of the cytokines IL-1β, IL-1RA, IL-17A, TNF-α, and G-CSF and the chemokines CCL7, CXCL1, CXCL8, CXCL11, and CXCL12α were found in comparison with BAL fluid of patients with influenza. IFN-γ, granzyme B, IL-6, IL-10, IL-15, IL-18, CCL2, CCL3, CCL4, CCL8, CXCL5, and CXCL10 levels were not significantly different from levels in BAL fluid of patients with influenza, although a tendency toward increased concentrations was seen in the patients with COVID-19. GM-CSF, IL-4, IL-5, IL-12p70, IL-12/IL-23p40, IL-23, and CCL11 were below the detection limit for most donors. Remarkable is the large variation seen among the different COVID-19 BAL samples. A positive correlation was found between COVID-19 BAL fluid levels of IL-15 and CXCL10 or CCL2, cytokines/chemokines involved in monocyte, lymphocyte, and NK cell functions (Figure 5, M and N). Moreover, a positive correlation was seen between levels of IL-1β or IL-17A and CXCL8 in the BAL fluid of the patients with COVID-19 (Figure 5, O and P). To conclude, the COVID-19 hypercytokinemia was associated with significantly elevated levels of cytokines and chemokines in the BAL fluid compared with the BAL fluid of influenza ARDS patients.

### Increased levels of protease inhibitors and similar net proteolytic activity in BAL fluid from COVID-19 compared with influenza patients.

**Figure 6:**
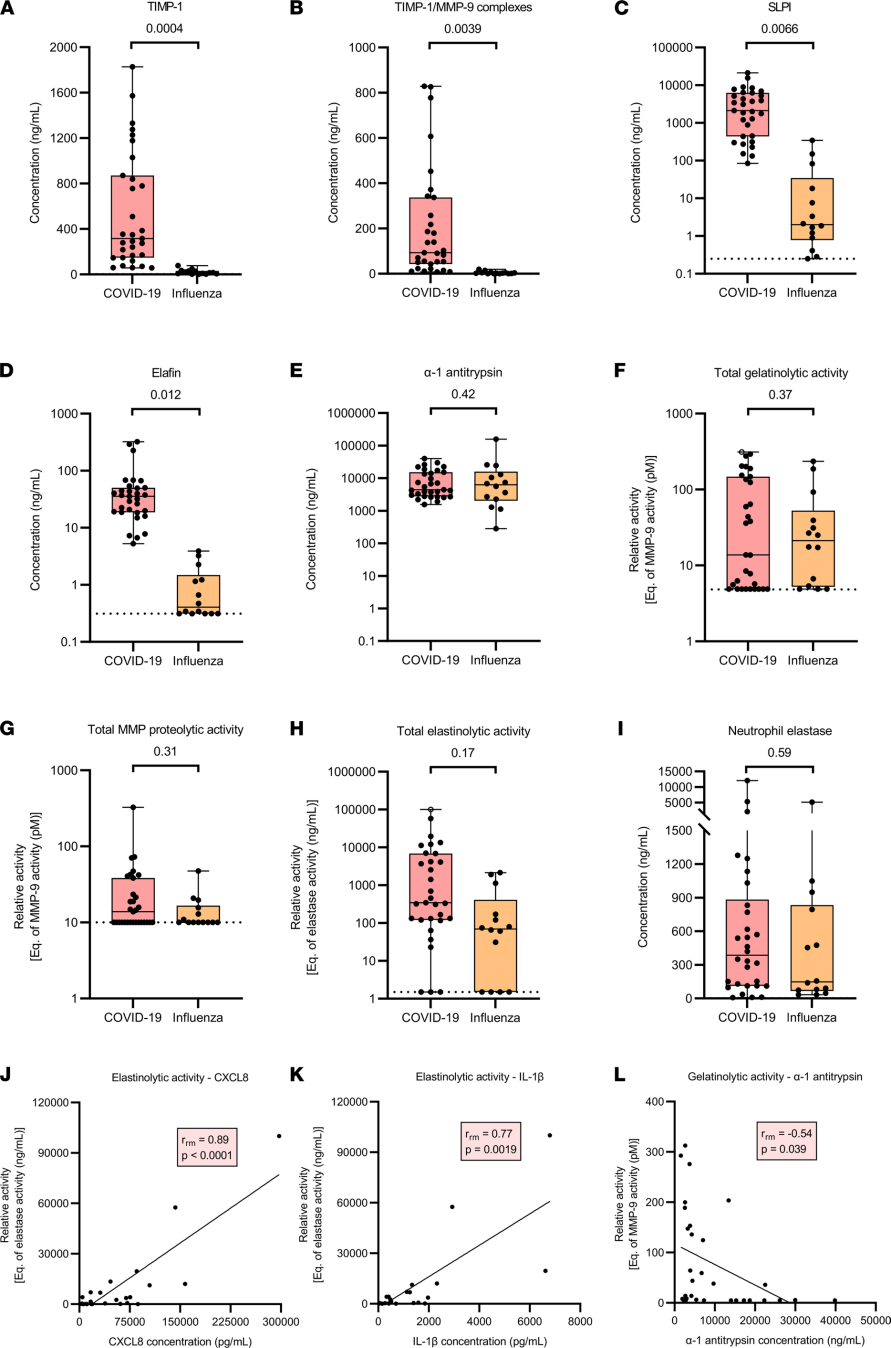
Quantification of protease activity and protease and protease inhibitor levels in BAL fluid from patients with severe COVID-19 or influenza. ELISA was used to determine concentrations of (**A**) TIMP-1, (**B**) TIMP-1/MMP-9 complexes, (**C**) SLPI, (**D**) elafin, and (**E**) α-1 antitrypsin (serpin A1) in BAL fluid samples from COVID-19 patients (*n* = 31) and influenza patients (*n* = 14). (**F**) Total gelatinolytic activity, as determined in a kinetic assay measuring degradation of a fluorogenic gelatin substrate; (**G**) total MMP proteolytic activity, as determined measuring degradation of a fluorogenic omni MMP substrate; (**H**) total elastinolytic activity, as determined measuring degradation of a fluorogenic elastin substrate; and (**I**) neutrophil elastase levels (quantified by ELISA) were measured within the BAL fluid samples. (**J**–**L**) Correlation between chemokine/cytokine levels, proteolytic activity, and protease inhibitors measured in the BAL fluid of COVID-19 patients. Data are shown as box-and-whisker plots (box: median with interquartile range, whiskers: full data distribution), with each dot representing an individual patient sample. The dashed lines indicate the lower detection limits. Unfilled symbols indicate values above the upper detection limit. Data were statistically analyzed by a linear mixed model with correction for multiple samples per patient using a random intercept model. Correlation analysis was performed calculating a repeated measures correlation coefficient and plotted utilizing a simple linear regression line. *P* values are shown above brackets.

Neutrophils store different proteases inside their granules, and these are released upon activation. However, a balance with protease inhibitors is crucial to prevent collateral damage to healthy (lung) tissues. Significantly increased levels of the metalloproteinase inhibitor tissue inhibitor of metalloproteinases 1 (TIMP-1) and of TIMP-1 in complex with MMP-9 were found in COVID-19 BAL fluids compared with influenza BAL fluids (Figure 6, A and B). Moreover, highly elevated levels of the locally produced serine protease inhibitors secretory leukocyte protease inhibitor (SLPI) and elafin were found in the BAL fluid of COVID-19 in comparison with influenza patients (Figure 6, C and D). In contrast, comparable levels of a major circulating serine protease inhibitor, α-1 antitrypsin (serpin A1), were detected in the BAL fluid of COVID-19 versus influenza patients (Figure 6E).

Due to the complex interactions between proteases and protease inhibitors, we measured the net proteolytic activity within the lungs. No significant differences in gelatinolytic activity or total MMP activity were found in the BAL fluid of COVID-19 versus influenza patients (Figure 6, F and G). However, a remarkably large variation was seen among the different patient samples. As we did not detect gelatinolytic activity within the parallel plasma samples (due to collection in tubes coated with EDTA), we applied the same analysis procedure on plasma samples (collected with tubes coated with citrate) from patients with COVID-19 in the ICU included in our previous study to enable comparison (8). For all these plasma samples, the relative activity is maximally equivalent to 39.3 pM MMP-9. For many samples, activities fell below the detection limit (estimated to be equivalent to 4.88 pM MMP-9) (Supplemental Figure 2, A and B). In some patient BAL samples, gelatinolytic activity was also below the detection limit, whereas other samples exhibited up to 50-fold higher activities (Figure 6F and Supplemental Figure 2, A and B). Comparable variability was observed for elastinolytic activity in the BAL fluids. A trend toward a 5-fold increase in median elastinolytic activity was seen in the BAL fluids of COVID-19 compared with influenza patients, but data did not reach significance (Figure 6H). By introducing protease inhibitors in the enzyme activity assays, we were able to assign the gelatinolytic and elastinolytic activities to both MMPs and serine proteases (Supplemental Figure 2, C–F). One of the major neutrophil proteases contributing to the degradation of elastin is the serine protease neutrophil elastase (28). However, no significant differences in neutrophil elastase concentrations were found between COVID-19 and influenza BAL fluids (Figure 6I). Interestingly, levels of IL-1β and CXCL8 in COVID-19 BAL fluid correlated positively with the elastinolytic and gelatinolytic activities measured (Figure 6, J and K; and Supplemental Figure 2, G and H). Finally, we uncovered a moderate but significant negative correlation between α-1 antitrypsin levels and gelatinolytic activity in the BAL fluid from patients with COVID-19, with higher concentrations of α-1 antitrypsin preventing severe proteolytic activity (Figure 6L). In conclusion, although high levels of metalloproteinase and serine protease inhibitors were detected in the BAL fluid of patients with COVID-19, the net proteolytic activity was not significantly altered compared with patients with influenza.

### High cytokine/chemokine levels persist in BAL fluid from patients with COVID-19 during antibiotic treatment for a bacterial coinfection.

**Figure 7:**
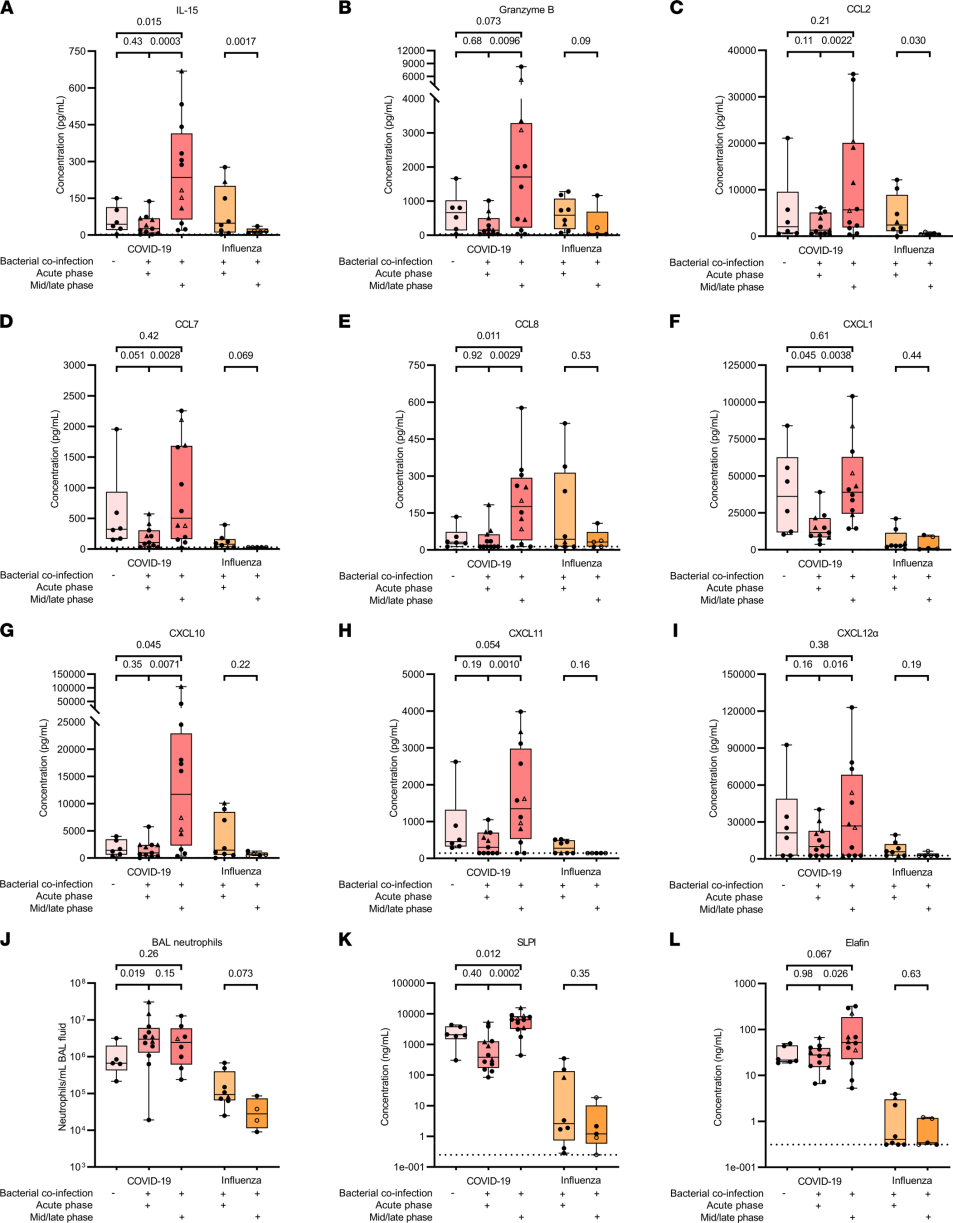
Quantification of biomarkers in BAL fluid from patients with severe COVID-19 or influenza, stratified by the timing of a bacterial coinfection. Multiplex and ELISA technology was used to determine concentrations of (**A**) IL-15, (**B**) granzyme B, (**C**) CCL2, (**D**) CCL7, (**E**) CCL8, (**F**) CXCL1, (**G**) CXCL10, (**H**) CXCL11, and (**I**) CXCL12α in COVID-19 and influenza BAL fluid samples. (**J**) BAL fluid neutrophil counts. (**K** and **L**) ELISA was used to determine (**K**) SLPI and (**L**) elafin concentrations in the BAL fluids. All COVID-19 samples were categorized based on the absence of a coinfection (*n* = 6) or the acute phase (*n* = 11) or mid/late phase of a bacterial coinfection (*n* = 12) based on the timing of the BAL sampling relative to the coinfection time course. Influenza patient samples were also categorized in the acute phase (*n* = 8) and the mid/late phase (*n* = 5) of a bacterial coinfection. Data are shown as box-and-whisker plots (box: median with interquartile range, whiskers: full data distribution), with each dot representing an individual patient sample. The dashed lines indicate the lower detection limits. In the mid/late groups, unfilled symbols indicate samples taken during the late phase of the bacterial coinfection; the others were taken during the midphase of the bacterial coinfection. Triangles indicate samples with an additional fungal coinfection. Data were statistically analyzed by a linear mixed model with correction for multiple samples per patient using a random intercept model or a Mann-Whitney *U* test, where appropriate. *P* values are shown above brackets.

Bacterial and fungal coinfections are common in COVID-19 ICU patients and are associated with a longer duration of ventilation (20, 21). Therefore, it was interesting to study the effect of coinfections on the inflammatory response. COVID-19 and influenza patient BAL samples were categorized based on the presence or absence of coinfection(s) and the type and timing of the coinfection(s). Coinfections were mostly of bacterial or combined bacterial-fungal origin (1 patient was diagnosed with a fungal coinfection only) in patients with COVID-19, whereas in patients with influenza all coinfections were bacterial with only 1 bacterial-fungal coinfection diagnosed (Figure 1, A and B). No significant differences were found in cytokine/chemokine levels (Supplemental Figures 3 and 4), protease activity, and levels of proteases and protease inhibitors (Supplemental Figure 5) in the BAL fluid of patients with COVID-19 with or without bacterial or combined bacterial-fungal coinfections. BAL fluid levels of TIMP-1/MMP-9 complexes were significantly elevated in COVID-19 patients having a bacterial-fungal coinfection compared with COVID-19 patients having a bacterial coinfection (Supplemental Figure 5B). However, for these interim analyses, samples were stratified solely based on the presence of a coinfection and the type of coinfection, without considering the timing of the coinfection. Therefore, we further subdivided the bacterial coinfections in acute phase (early phase of coinfection with clinical/biochemical worsening and antibiotics not yet or recently started), midphase (signs of improvement with ongoing antibiotic therapy), or late phase (final days of antibiotic therapy nearing complete remission) based on the timing of the BAL sample analyzed relative to the coinfection time course (Figure 7). Concentrations of IL-15, granzyme B, CCL2, CCL7, CCL8, CXCL1, CXCL10, CXCL11, and CXCL12α, i.e., inflammatory mediators associated with attraction or activation of monocytes, lymphocytes, and NK cells, were significantly increased in the BAL fluid of patients with COVID-19 in the mid- or late phase compared with patients in the acute phase of the coinfection (Figure 7, A–I). IL-15, CCL8, and CXCL10 levels were also significantly elevated in the BAL fluid of patients with COVID-19 in the mid- or late phase of a coinfection compared with patients without coinfections. Thus, despite treatment with antibiotics, the highest BAL fluid concentrations of these cytokines/chemokines in patients with COVID-19 were detected in later phases of the bacterial coinfection. This contrasts with the influenza BAL samples, for which these cytokine/chemokine levels tended to be lower in later phases of a bacterial coinfection compared with the acute phase. Neutrophil counts in the BAL fluid of patients with COVID-19 having an acute coinfection were significantly increased compared with COVID-19 patients without coinfection. Furthermore, neutrophil counts did not become lower upon treatment with antibiotics, in contrast with patients with influenza, in whom a trend for reduction was seen (Figure 7J). In addition, significantly increased BAL fluid levels of the protease inhibitors SLPI and elafin were found during the mid/late phase compared with the acute phase of the coinfection in patients with COVID-19 (but not with influenza) (Figure 7, K and L). However, in patients with COVID-19, this did not correlate to significant changes in proteolytic activity or other protease/protease inhibitor levels during different phases of the coinfection (Supplemental Figure 6). In addition, levels of the other cytokines, mononuclear leukocyte-derived CCL3 and CCL4, and the neutrophil attractant chemokines CXCL5 and CXCL8 were not significantly different during different phases of the coinfection (Supplemental Figure 7 and Supplemental Figure 8, A–D). No significant differences were noticed in the timing (days after ICU admission) of the BAL sampling (Supplemental Figure 8E) or the SARS-CoV-2 and influenza viral load (Supplemental Figure 8F) in the BAL samples from patients with COVID-19 and with influenza without coinfection, or from patients in the acute or mid/late phase of a bacterial coinfection. Moreover, no correlations were found between the cytokine/chemokine levels and the viral load in the BAL samples, excluding an exclusively viral effect on the elevated inflammatory mediators during the later phases of the bacterial coinfection. In conclusion, despite antibiotic treatment for bacterial coinfections, critically ill COVID-19 patients maintained very high levels of IL-15, granzyme B, CCL2, CCL7, CCL8, CXCL1, CXCL10, CXCL11, and CXCL12α and the serine protease inhibitors SLPI and elafin in their lungs, whereas after influenza infection these molecules returned to basal levels in the recovery phase. This suggests that bacterial coinfection triggers a stronger and more long-lasting inflammatory response in patients with COVID-19, even during treatment with antibiotics and corticosteroids.

## Discussion

It is now well established that an atypical cytokine storm drives the systemic inflammation in severe COVID-19 (3, 4, 12, 29, 30), which is confirmed by our results. In the BAL fluid of COVID-19 compared with critically ill influenza patients, we detected elevated and extremely high levels of the cytokines IL-1β, IL-1RA, IL-17A, TNF-α, and G-CSF and the chemokines CCL7, CXCL1, CXCL8, CXCL11, and CXCL12α, expanding earlier reports showing increased concentrations of inflammatory mediators compared with BAL fluid of healthy donors or patients with moderate influenza or COVID-19 (30–32). Single-cell transcriptomics and flow cytometry studies on the BAL fluid of patients with COVID-19 show elevated numbers of proinflammatory monocyte-derived macrophages in severe cases compared with cases that were rather moderate or non–COVID-19 pneumonia (17, 18, 32–34). These macrophages represent a potentially important source of proinflammatory mediators. Considering the discovery of CXCL8 as an IL-1β–induced protein (35), we found that COVID-19 BAL fluid levels of CXCL8 and IL-1β correlated positively with each other and with levels of IL-17A. Clonally expanded tissue-resident memory-like Th17 cells, with expression of *IL17A* in the lungs, and elevated IL-17A levels in the BAL fluid, were detected in patients with severe COVID-19 (36). Evidence for a crosstalk between human neutrophils and Th17 cells was already shown (37), along with additional evidence for neutrophils promoting the induction of Th17 cells in patients with COVID-19 (38).

The 10- to 100-fold higher levels of the most potent human neutrophil-attracting chemokine, CXCL8, and the neutrophil-attracting chemokines CXCL1 and CXCL5 in the BAL fluid of COVID-19 versus influenza patients could explain the major neutrophil infiltration in the lungs. Lung neutrophils displayed a hyperactivated phenotype, in comparison with the already activated neutrophils in the blood, as evidenced by near-complete shedding of L-selectin; downregulation of CD16, CXCR1, CXCR2, and C5aR; and upregulation of CD11b, CD11c, CD49d, FPR1, FPR2, CD32, CD64, CD69, CD14, CD66b, CD15, and CD63. Moreover, NETs have been previously detected in the airway, interstitial, and vascular compartments of the lungs of patients with COVID-19 (39). It has been proposed that a self-sustaining positive feedback loop of systemic and neutrophil-intrinsic CXCL8 production could lead to an activated, prothrombotic neutrophil phenotype characterized by degranulation and NET formation (40). Interestingly, a significant portion of the BAL fluid neutrophils expressed CXCR4 (Figure 3C), a receptor present on immature neutrophils in the bone marrow and shown to reappear on aged neutrophils (27). Expression of *CXCR4* by neutrophils in patients with COVID-19 was already shown by single-cell RNA sequencing in the lungs (10). Based on (absence of) coexpression of CD10 and CD49d, it seemed that both immature as well as aged neutrophils are present in the BAL fluid. The immature neutrophils might represent the “progenitor” neutrophils already found (18). Finally, some neutrophils upregulated antigen-presenting molecules HLA-DR and HLA-DQ within the BAL fluid. Such neutrophils can actively present antigens to T cells, potentially playing a role in the regulation of adaptive immunity (41). Interestingly, these “hybrid” neutrophils were previously found in BAL fluid by single-cell RNA sequencing (18).

Upon activation, neutrophils release several proteases, protease inhibitors, and antimicrobial proteins into the extracellular environment (7, 42). In patients with ARDS, continuous proteolytic damage can cause sustained inflammatory cell infiltration, progressive lung tissue damage, fibrin deposition, and hyaline membrane formation and in some cases triggers fibroblast activation and fibrosis (43). Indeed, diffuse alveolar damage is a common characteristic seen in postmortem histopathological lung analysis from patients who died from COVID-19 (44). Elastin-degrading (and other extracellular matrix protein–degrading) proteases released by neutrophils could contribute to alveolar damage, resulting in protein-rich alveolar edema (45, 46). Neutrophil elastase, proteinase 3, cathepsin G, and activated cathepsin C have been detected in endotracheal aspirates of mechanically ventilated patients with ARDS associated or not associated with COVID-19 (47). We did not measure significant differences in neutrophil elastase concentrations between COVID-19 and influenza BAL fluid. However, neutrophil elastase concentrations were much higher in comparison with the concentrations we found in COVID-19 plasma samples (8). In addition, we detected 100- to 1000-fold higher levels of SLPI and elafin in the BAL fluid of COVID-19 in comparison with influenza patients. These serine protease inhibitors are produced at mucosal surfaces in the lungs by epithelial cells and leukocytes, including neutrophils, and provide a local inducible antiprotease and antiinflammatory safeguard (48). Despite these high levels of protease inhibitors in patients with COVID-19, the net proteolytic activity in the lungs was not significantly altered compared with patients with influenza, indicative of concomitant high protease levels. Follow-up research is required to unveil the putative roles played by proteases other than neutrophil elastase in the pathology of COVID-19 versus influenza. The abundantly present protease inhibitors in the COVID-19 patient lungs could also be inactivated by proteolytic cleavage and still remain detectable by ELISA. For the protease inhibitor SLPI, it has been shown that MMP-9 and other neutrophil-derived proteases can cleave SLPI, resulting in a reduced capacity to inhibit neutrophil elastase activity (49).

The circulating, neutrophil elastase–targeting, acute-phase protein α-1 antitrypsin (serpin A1) was abundantly present in the BAL fluid of both COVID-19 and influenza patients. Neutrophil elastase can contribute to proteolysis of the SARS-CoV-2 glycoproteins allowing membrane fusion in the host (50). Therefore, by inhibiting neutrophil elastase, α-1 antitrypsin may impair SARS-CoV-2 infection. Besides, α-1 antitrypsin was shown to inhibit transmembrane serine protease 2, the protease priming the SARS-CoV-2 spike protein for entry into host cells (51). We showed a negative correlation between α-1 antitrypsin levels and gelatinolytic activity in the BAL fluid of the patients with COVID-19, with higher concentrations of α-1 antitrypsin preventing severe proteolytic activity. Therefore, for patients having high proteolytic activity within the lungs, the use of inhibitors targeting neutrophil-derived proteases might be a useful additional treatment strategy to prevent excessive proteolytic damage. As IL-1β and CXCL8 levels in the BAL fluid correlated to the proteolytic activity, such high levels could be a relevant indication for treatment. Interestingly, BAL fluid levels of CXCL8 were specifically shown to be predictive for COVID-19 severity and may also serve as a potential biomarker for predicting COVID-19 progression (30). Due to both its antiviral and antiinflammatory role, α-1 antitrypsin was already suggested as a good candidate for treatment of COVID-19 ARDS (52–54). Currently, several clinical trials are ongoing evaluating the use of α-1 antitrypsin and neutrophil-derived protease inhibitors for COVID-19 treatment (NCT04385836, NCT04547140, NCT04495101, NCT04817332; ClinicalTrials.gov). In short, hospitalized COVID-19 patients are being recruited for phase II clinical trials for efficacy evaluation of α-1 antitrypsin (either by intravenous injection or inhalation) aiming to reduce mortality or requirement of intensive care. Moreover, a phase III clinical trial is investigating the potential of brensocatib (INS1007) as a novel therapy for adult patients hospitalized with COVID-19. Brensocatib is an oral reversible inhibitor of dipeptidyl peptidase 1, an enzyme responsible for activation of neutrophil serine proteases. Interestingly, brensocatib has been shown to reduce neutrophil serine protease activity and improves clinical outcomes in patients with bronchiectasis (55).

The presence of coinfections in the lungs could have an important influence on disease outcomes because it was shown that coinfections are associated with a longer duration of ventilation in critically ill COVID-19 patients (20, 21). It was proposed that the COVID-19 cytokine storm may be the result of synergistic interactions among Toll-like receptors and nucleotide-binding oligomerization domain-like receptors due to combined infections of SARS-CoV-2 and other microbes (56). When stratifying bacterial coinfections based on the timing of the BAL sample analyzed relative to the coinfection time course, we found that BAL fluid levels of several inflammatory proteins acting on monocytes, lymphocytes, and NK cells in patients with COVID-19 persisted or even increased beyond the acute phase of a coinfection, when patients remained on antibiotic treatment. This contrasts with the (trend for a) drop in release of these mediators in antibiotic-treated ICU influenza patients with coinfections. As viral loads were comparable in the COVID-19 BAL samples taken during the acute or later phases of a bacterial coinfection, we hypothesize that it is this second bacterial stimulus and the synergy between SARS-CoV-2 and bacterial pathogen-associated molecular patterns that prevent the reduction of these inflammatory mediators in the long term, during antibiotic and corticosteroid treatment. Together with the diminished type I and type III IFN production (untuned antiviral immunity) in COVID-19 in comparison with influenza (57) and defects in the sensing of viral RNA (inborn errors in type I IFN immunity) in some patients with life-threatening COVID-19 (58), this may at least partially account for the prolonged stay and higher mortality of patients with COVID-19 in the ICU. Given the many clinical trials investigating the use of cytokine-modulating therapies for COVID-19 treatment, it would be worthwhile to study the influence of these therapies on the inflammatory response to coinfections and the antimicrobial treatment.

This study has some unavoidable limitations. First, since no influenza patients were treated in our university hospital during the COVID-19 pandemic, we could not collect influenza BAL neutrophils for flow cytometric analysis and were restricted to the analysis of influenza BAL fluid collected during the previous winter. Second, the number of saline aliquots used during BAL sampling was slightly different between patients with influenza and with COVID-19. The amount of fluid recovered from the total volume instilled was also slightly different for every patient. These are well-known limitations of BAL fluid sampling for which it is difficult to correct and that might have consequences regarding protein concentrations or activity. However, BAL sampling was always performed on the same location in the lungs and with the same volume of saline per aliquot, ensuring that comparable areas in the lungs were included. Due to the observed specific differences between COVID-19 and influenza patients (for certain parameters up to 1000-fold, with other factors being similar), we are confident that the comparisons we have made are reliable. Third, our sample size is limited, which is mainly due to practical limitations that come with the analysis of neutrophils and BAL fluids (use of a biosafety level 3 [BSL3] facility and ethical permission). Fourth, no time component was included in our analysis. Some cytokine, chemokine, and protease inhibitor levels tended to be lower in COVID-19 patient BAL samples collected later during ICU stay compared with samples collected earlier during ICU stay (Supplemental Figure 9). However, for other cytokines and chemokines, this trend was not visible, and since the clinical situation of the patients with COVID-19 patients was highly variable during hospital stays, an adequate analysis of data kinetics was difficult. Moreover, the COVID-19 BAL samples collected early during ICU stay would be most “comparable” to the influenza BAL samples with respect to BAL sampling timing. The differences between these early COVID-19 and influenza patient samples were even more pronounced for certain biomarkers. Moreover, timing of BAL sample collection did not influence the coinfection data significantly (Supplemental Figure 8E). Finally, there might be other unavoidable factors confounding our analysis: patients had divergent comorbidities, were on different therapies, received artificial ventilation, and had different coinfections.

In conclusion, we show hyperinflammation characterized by significantly increased cytokine and chemokine levels, hyperactivated neutrophils, and elevated levels of protease inhibitors TIMP-1, SLPI, and elafin in the lungs of critically ill COVID-19 patients in comparison with influenza patients. In contrast to patients with influenza, the cytokine storm in the lungs persisted or even increased during antimicrobial treatment for a bacterial coinfection in patients with COVID-19. This suggests that synergy between bacterial coinfections and SARS-CoV-2 triggers a stronger production of these inflammatory mediators in the long term, despite antibiotic and corticosteroid treatment, which may at least partially account for the prolonged stay at the ICU of COVID-19, in comparison with influenza, patients.

## Methods

### Study design.

Seventeen critically ill adult patients with COVID-19 were recruited at the University Hospital Leuven between November and December 2020. All patients were on invasive mechanical ventilation or received ECMO in the ICU. In total, 31 fresh blood and parallel BAL samples were collected from the patients with COVID-19 in a period 4–37 days after ICU admission, upon clinical indication (Figure 1A). Blood samples from age- and sex-matched healthy individuals were investigated for comparative purposes. Healthy individuals were recruited from Rega Institute staff and the University Hospital Leuven. Measurements were compared with stored BAL supernatant of 14 adult patients with influenza with invasive mechanical ventilation as the minimum level of respiratory support (except for 2 samples) collected at 4–6 days after ICU admission in the influenza season of 2019 and 2020 (Figure 1B). The objectives of this study were (a) to characterize the phenotype of BAL fluid and parallel blood neutrophils in critically ill COVID-19 ICU patients and compare this with blood neutrophils of healthy controls; (b) to determine the levels of inflammatory cytokines, chemokines, proteases, and protease inhibitors within the plasma and BAL fluid of COVID-19 patients and compare them with influenza patients as a non–COVID-19 viral pneumonia control group; and (c) to study the effect of a bacterial or fungal coinfection(s) in this context.

### Assessment of coinfections.

Two clinicians assessed the presence of bacterial and fungal coinfection(s) independently. Biochemical and microbial test culture results were evaluated in combination with clinical and radiological characteristics to detect all clinically relevant coinfections. Bacterial coinfection was scored as acute phase (early phase of coinfection with clinical/biochemical worsening and antibiotics not yet or recently started), midphase (signs of improvement with ongoing antibiotic therapy), or late phase (final days of antibiotic therapy nearing complete remission) based on the timing of BAL sample analyzed relative to the coinfection time course. Diagnosis of probable invasive pulmonary aspergillosis was based on radiological abnormalities in combination with clinical signs and mycological evidence (positive galactomannan in BAL and/or serum and/or presence of *Aspergillus fumigatus* in BAL culture) as defined by Koehler et al. (59).

### Processing of blood and BAL samples.

Fresh blood and BAL samples were processed within 30 minutes of withdrawal. Blood samples were collected in vacutainer tubes (BD Biosciences) treated with EDTA. Blood samples were spun down for 10 minutes at 400*g*. The supernatant was collected and centrifuged for 20 minutes at 16,000*g* to obtain platelet-free plasma. BAL samples from patients with COVID-19 were collected via bronchoscopy by instilling 2 aliquots of 20 mL sterile saline in the right middle lobe or lingula, after which the returned fractions were immediately pooled for further processing. BAL samples from patients with influenza were collected by instilling 3–5 aliquots of 20 mL sterile saline in the right middle lobe or lingula. BAL samples from patients with COVID-19 were processed in the BSL3 facility of the Rega Institute, KU Leuven. BAL samples were centrifuged for 8 minutes at 500*g* at room temperature to collect BAL supernatant. Plasma and BAL supernatants were stored until further use at –80°C. To collect cells for flow cytometry, the cell pellet was resuspended 1:1 in 0.1% dithiothreitol, vortexed for 15 minutes, and filtered through a nylon filter (Falcon 40 μm cell strainer, Corning) to remove excess mucus. After centrifugation for 8 minutes at 500*g* at room temperature, the supernatants were discarded, and the pellets were resuspended in Dulbecco’s PBS for counting.

### Isolation of neutrophils.

Blood neutrophils used for phenotypical characterization were isolated from the whole peripheral blood by immuno-magnetic negative selection according to the manufacturer’s instructions (EasySep Direct Human Neutrophil Isolation Kit; STEMCELL Technologies) within 30 minutes of withdrawal.

### Phenotypical analysis of neutrophils.

Neutrophil phenotyping was performed on isolated blood neutrophils and the BAL cell pellet (without previous neutrophil purification). Cells were treated with Fc receptor block (Miltenyi Biotec) and Fixable Viability Stain 620 (BD Biosciences) or Zombie Aqua 516 (BioLegend) for 15 minutes at room temperature. Subsequently, cells were washed with flow cytometry buffer (PBS + 2% *v/v* FCS + 2 mM EDTA) and stained with fluorescently labeled antibodies. Antibodies used in this study were titrated in-house and are listed in Supplemental Table 1. Following incubation for 25 minutes (on ice), cells were washed with flow cytometry buffer and fixed with BD Cytofix (BD Biosciences). Results were analyzed using a BD LSRFortessa X-20 (BD Biosciences) equipped with Diva software (BD Biosciences). FlowJo software (BD Biosciences) was used for downstream analysis. Neutrophils were gated as CD16^+^CD66b^+^ cells within the population of living single cells (Supplemental Figure 10).

### Quantification of cytokines, chemokines, proteases, and protease inhibitors.

Plasma and BAL supernatant concentrations of IL-1β, IL-1RA, IL-4, IL-5, IL-6, IL-10, IL-12p70, IL-12/IL-23p40, IL-15, IL-17A, IL-18, IL-23, IFN-γ, TNF-α, G-CSF, GM-CSF, granzyme B, CXCL5, CXCL10, CXCL11, CXCL12α, CCL2, CCL3, CCL4, CCL7, CCL8, and CCL11 were measured using customized Meso Scale Discovery multiplex assays. CXCL8 concentrations in BAL were evaluated using a specific sandwich ELISA developed in our laboratory (8). CXCL1, neutrophil elastase, TIMP-1, TIMP-1/MMP-9 complexes, SLPI, Serpin A1, and Trappin-2/Elafin were quantified by DuoSet ELISAs (R&D Systems) in BAL supernatant.

### Measurement of elastinolytic, gelatinolytic, and MMP activity.

To measure gelatinase or metalloproteinase activity, 15 μL of dye-quenched gelatin (DQ-gelatin, Thermo Fisher Scientific) (final concentration of 5 μg/mL) or OmniMMP substrate peptide (Mca-PLGL-Dpa-AR-NH_2_, catalog BML-P126-0001, Enzo Life Sciences) (final concentration of 5 μg/mL) in assay buffer (50 mM Tris, 150 mM NaCl, 5 mM CaCl_2_, 0.01% Tween-20, pH 7.4) was added to 5 μL BAL supernatant, respectively. A standard series was created by preparing serial dilutions of activated recombinant MMP-9, produced as previously described (60). To measure elastinolytic activity, 15 μL of DQ-elastin (Thermo Fisher Scientific) (final concentration of 15 μg/mL) in Tris-HCl buffer (0.1 M, pH 8.0) was added to 5 μL BAL supernatant. A standard series was created by elastase dilutions (elastase from pig pancreas, Thermo Fisher Scientific). Fluorescence was measured over time with the CLARIOstar microplate reader (BMG Labtech) for 1 hour at 37°C. Metalloproteinase activity and serine protease activity were inhibited by the addition of EDTA (125 mM) or 4-(2-aminoethyl)-benzene-sulfonyl fluoride (1 mg/mL, Pefabloc SC, Merck), respectively. The slopes of the kinetic curves were determined, and all data shown are represented as the equivalent of standard enzymatic activity.

### Statistics.

No normal distribution of data was detected as evaluated by the Shapiro-Wilk test. Mann-Whitney *U* tests were used to statistically compare COVID-19 (*n* = 17) and influenza (*n* = 14) patient characteristics. A linear mixed model was used to detect statistical differences within and between COVID-19 (*n* = 31) and influenza (*n* = 14) BAL and blood samples. Correction for multiple samples per patient was done using a random intercept model. Statistical tests for comparison were 2 sided, and *P* < 0.05 was considered significant. For data values below the lower detection limit, half the value of the lower detection limit was used for statistical comparison. The central lines in the boxes of the box-and-whisker plots represent the median, while the bounds of the boxes represent the interquartile range, with the whiskers indicating the full distribution of the data. All outliers were included in the data and all data points are shown. Correlation analysis was performed by calculating a repeated measures correlation coefficient with the rmcorr function in R and plotted utilizing a simple linear regression line. Statistical analysis was performed using RStudio version 1.4, and GraphPad Prism 9 (GraphPad Software) was employed for visualization of the data.

### Study approval.

Written informed consent was obtained from all study participants or their legal representatives according to the ethical guidelines of the Declaration of Helsinki. The Ethics Committee of the University Hospitals Leuven approved this study (S63881).

## Author contributions

SC, MM, DS, P Matthys, GO, JW, JV, and PP designed the experiments. SC, MM, ACDC, CJ, LV, BMD, MG, JV, and PP developed methodology for the experiments and data analysis. SC, MM, ACDC, AN, CJ, LV, BMD, EH, and JW performed experiments and analyzed data. CJ, LV, P Meersseman, GH, EW, AW, and JW were involved in clinical data and patient sample collection. SC, MM, AN, CJ, and BMD visualized the data. MG, Kim Martinod (member of the CONTAGIOUS Consortium), P Matthys, GO, REM, JW, JV, and PP acquired funding for this study. DS, P Matthys, GO, REM, JW, JV, and PP supervised this study. SC wrote the original draft of this manuscript, and all authors reviewed, edited, and approved the final version of the manuscript.

## Supplementary Material

Supplemental data

## Figures and Tables

**Table 2:**
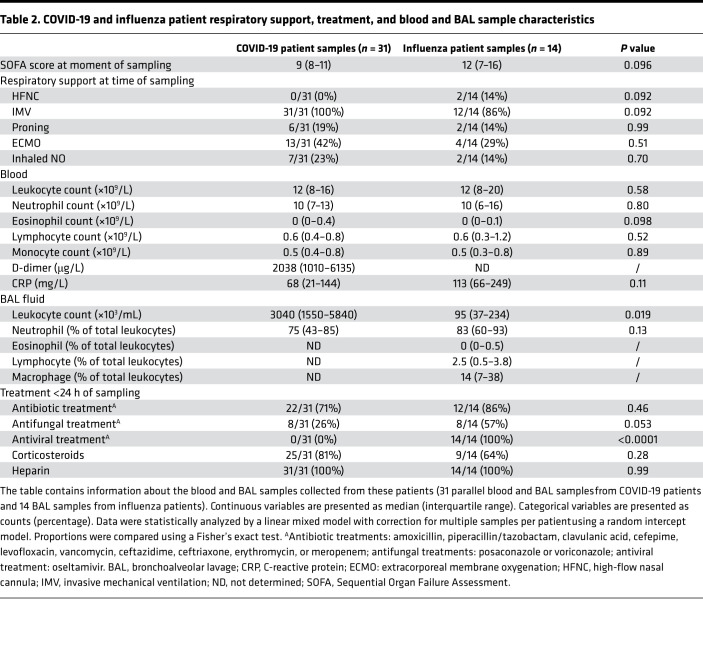
COVID-19 and influenza patient respiratory support, treatment, and blood and BAL sample characteristics

**Table 1:**
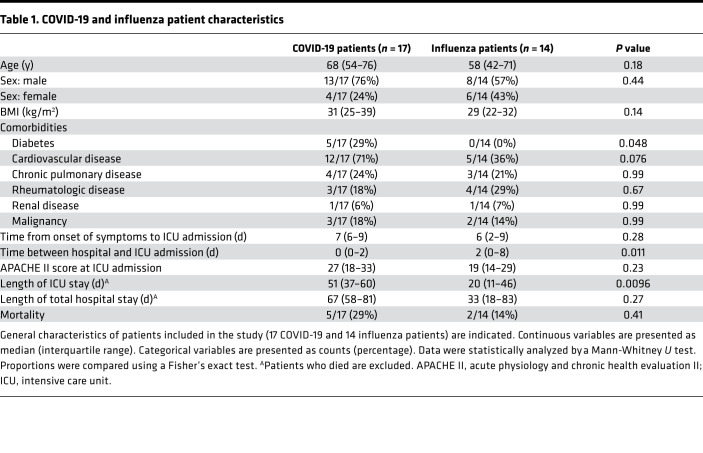
COVID-19 and influenza patient characteristics

## Version 1 11/18/2021

In-Press Preview

## Version 2 01/11/2022

Electronic publication
