# Supplementary material for: Atypical response to bacterial coinfection and persistent neutrophilic bronchoalveolar inflammation distinguish critical COVID-19 from influenza
Source: JCI Insight. 2022 Jan 11;7(1):e155055. doi: 10.1172/jci.insight.155055 (PMC8765057; doi:10.1172/jci.insight.155055)
Supplement: Supplemental data [file jciinsight-7-155055-s051.pdf]

SUPPLEMENTAL FIGURES & TABLES

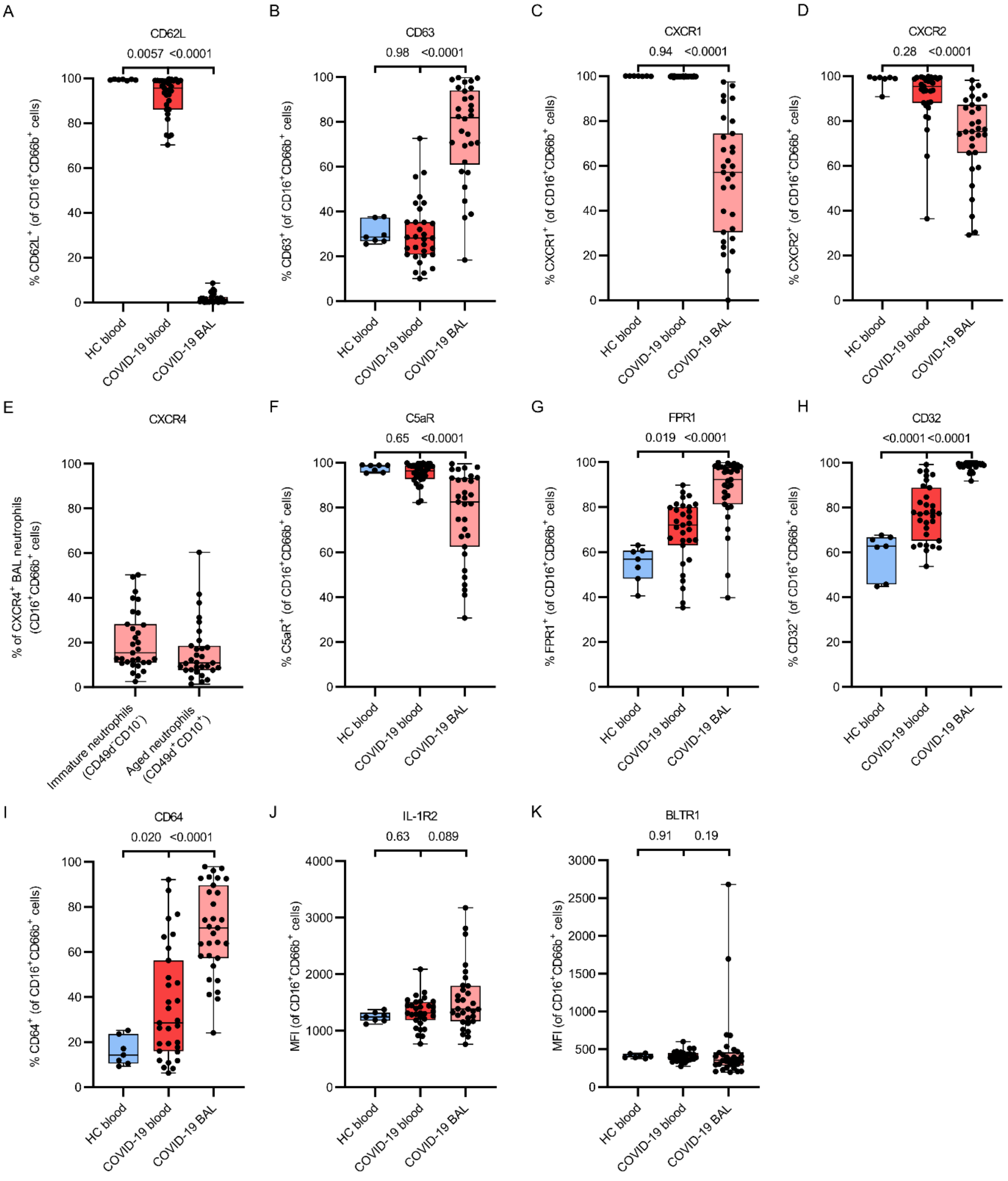

Supplemental Figure 1. Phenotypical characterization of membrane markers on BAL fluid and peripheral blood neutrophils from patients with severe COVID-19. Flow cytometry was used to evaluate the surface expression of **(A)** CD62L, **(B)** CD63, **(C)** CXCR1, **(D)** CXCR2, **(E)** CXCR4 in combination with CD49d and CD10 to define immature or aged neutrophils, **(F)** C5aR, **(G)** FPR1, **(H)** CD32, **(I)** CD64, **(J)** IL-1R2 and **(K)** BLTR1 on neutrophils (gated as $CD16^{+}CD66b^{+}$ cells) from paired blood and BAL fluid samples from COVID-19 patients (n = 31) and blood samples from healthy controls (HC) (n = 7). Results represent percentages of positive neutrophils or median fluorescence intensity (MFI). Data are shown as box-and-whisker plots (box: median with interquartile range, whiskers: full data distribution) with each dot representing an individual patient sample and statistically analyzed by a linear mixed model with correction for multiple samples per patient using a random intercept model.

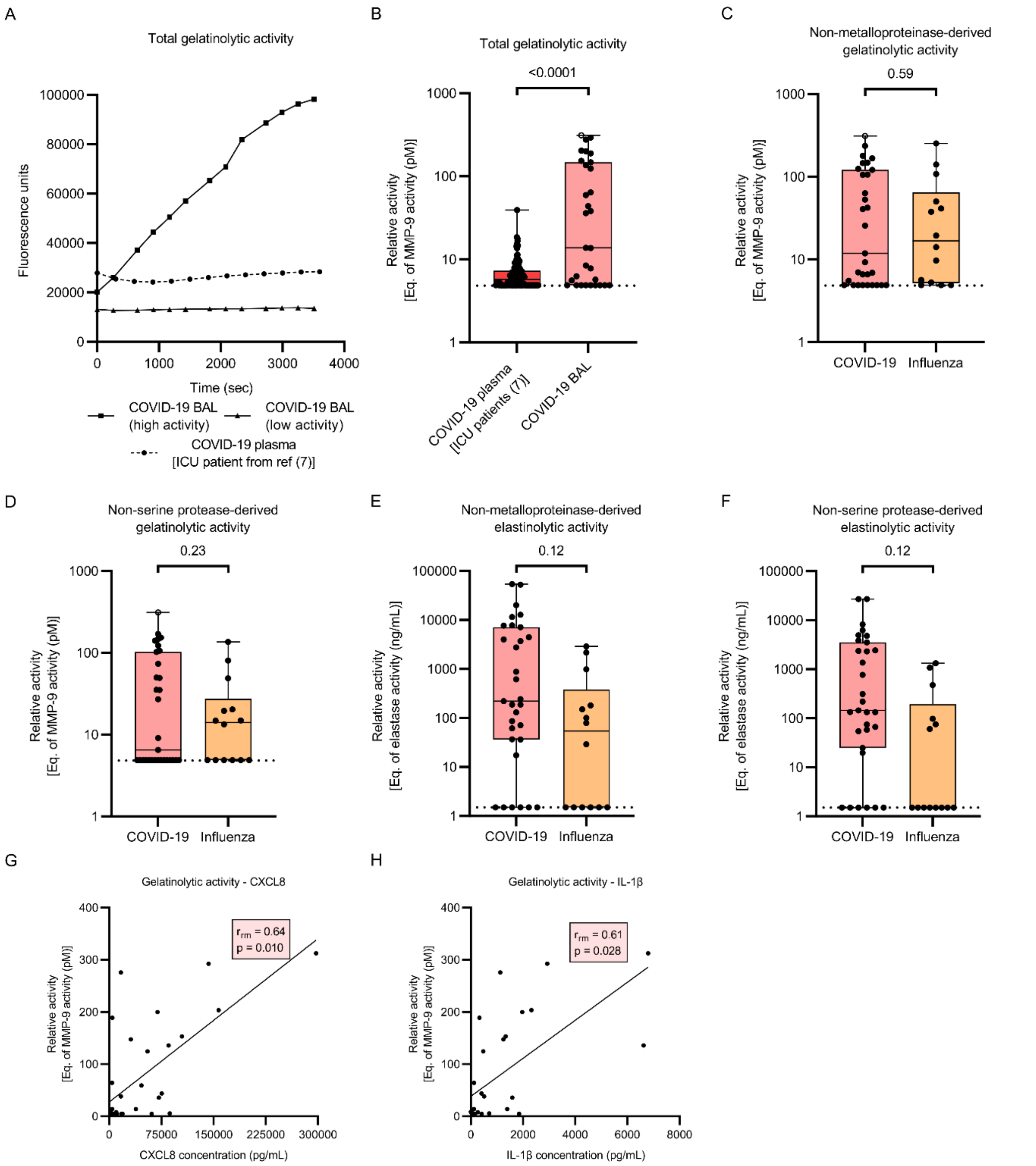

Supplemental Figure 2. Quantification of protease activity in BAL fluid and plasma from patients with severe COVID-19 or influenza. (A) Total gelatinolytic activity, as determined in a kinetic assay measuring degradation of a fluorogenic gelatin substrate, showing the fluorescence increase in function of time for two representative COVID-19 BAL samples (one with high and one with low activity) and a COVID-19 plasma sample from a previously published ICU patient cohort without BAL fluid collection (7). **(B)** Total gelatinolytic activity in COVID-19 plasma samples from the same ICU patient cohort (all samples are collected from patients at ICU admission, after one week or at ICU discharge, as explained in (7), *n* = 114) and COVID-19 BAL samples (*n* = 31). **(C-D)** Gelatinolytic activities, **(C)** in the presence of the metalloproteinase inhibitor EDTA or **(D)** the serine protease inhibitor AEBSF, were determined in BAL fluid samples from COVID-19 patients (*n* = 31) and influenza patients (*n* = 14). **(E-F)** Elastinolytic activities, as determined in a kinetic assay measuring degradation of a fluorogenic elastin substrate, **(E)** in the presence of EDTA or **(F)** AEBSF were determined within the BAL fluid samples. **(G-H)** Correlation between CXCL8 or IL-1β levels and gelatinolytic activity measured in the BAL fluid of COVID-19 patients. Data are shown as box-and-whisker plots (box: median with interquartile range, whiskers: full data distribution) with each dot representing an individual patient sample. The dashed lines indicate the lower detection limits. Open symbols indicate values above the upper detection limit. Data were statistically analyzed by a linear mixed model with correction for multiple samples per patient using a random intercept model. Correlation analysis was performed calculating a repeated measures correlation coefficient and plotted utilizing a simple linear regression line.

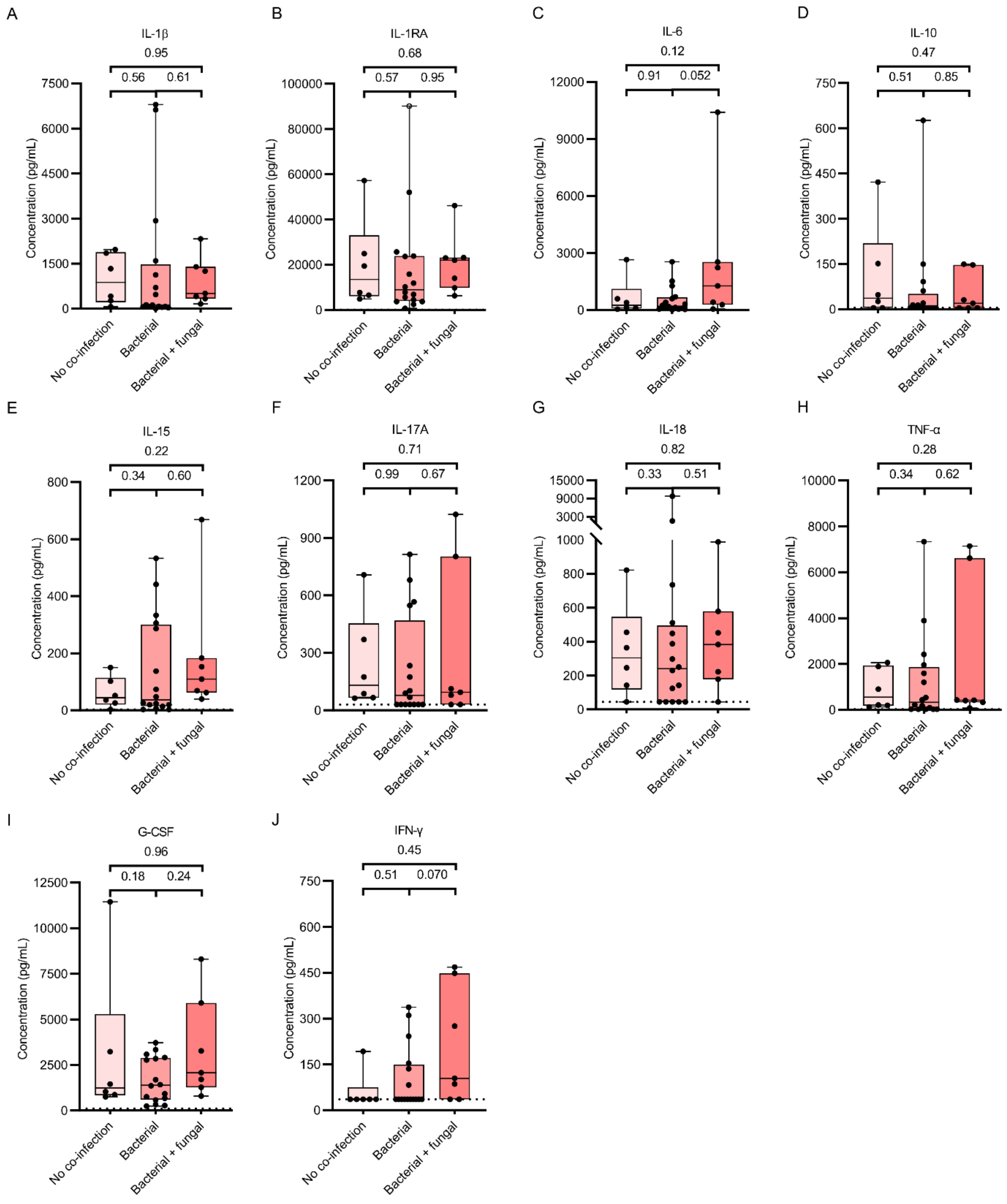

Supplemental Figure 3. Quantification of cytokines in BAL fluid from patients with severe COVID-19, stratified by the presence and type of a co-infection. Multiplex technology was used to determine concentrations of **(A)** IL-1β, **(B)** IL-1RA, **(C)** IL-6, **(D)** IL-10, **(E)** IL-15, **(F)** IL-17A, **(G)** IL-18, **(H)** TNF-α, **(I)** G-CSF and **(J)** IFN-γ in BAL fluid samples from COVID-19 patients having no co-infection (n = 6) or having a bacterial (n = 16) or combined bacterial-fungal co-infection (n = 7). Data are shown as box-and-whisker plots (box: median with interquartile range, whiskers: full data distribution) with each dot representing an individual patient sample. The dashed lines indicate the lower detection limits. Open symbols indicate values above the upper detection limit. Data were statistically analyzed by a linear mixed model with correction for multiple samples per patient using a random intercept model.

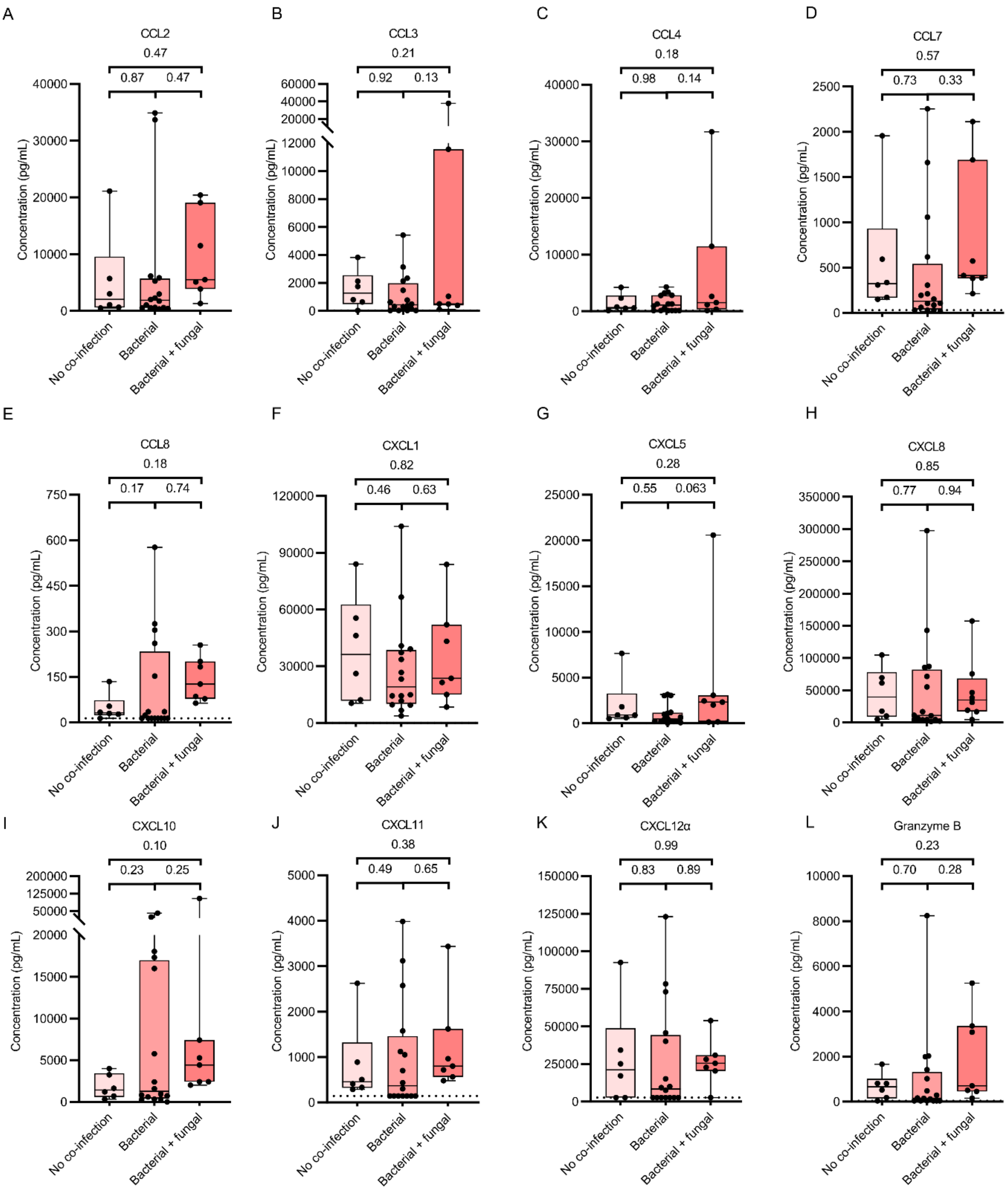

Supplemental Figure 4. Quantification of biomarkers in BAL fluid from patients with severe COVID-19, stratified by the presence and type of a co-infection. Multiplex and ELISA technology was used to determine concentrations of **(A)** CCL2, **(B)** CCL3, **(C)** CCL4, **(D)** CCL7, **(E)** CCL8, **(F)** CXCL1, **(G)** CXCL5, **(H)** CXCL8, **(I)** CXCL10, **(J)** CXCL11, **(K)** CXCL12α and **(L)** granzyme B in BAL fluid samples from COVID-19 patients having no co-infection (n = 6) or having a bacterial (n = 16) or combined bacterial-fungal co-infection (n = 7). Data are shown as box-and-whisker plots (box: median with interquartile range, whiskers: full data distribution) with each dot representing an individual patient sample. The dashed lines indicate the lower detection limits. Data were statistically analyzed by a linear mixed model with correction for multiple samples per patient using a random intercept model.

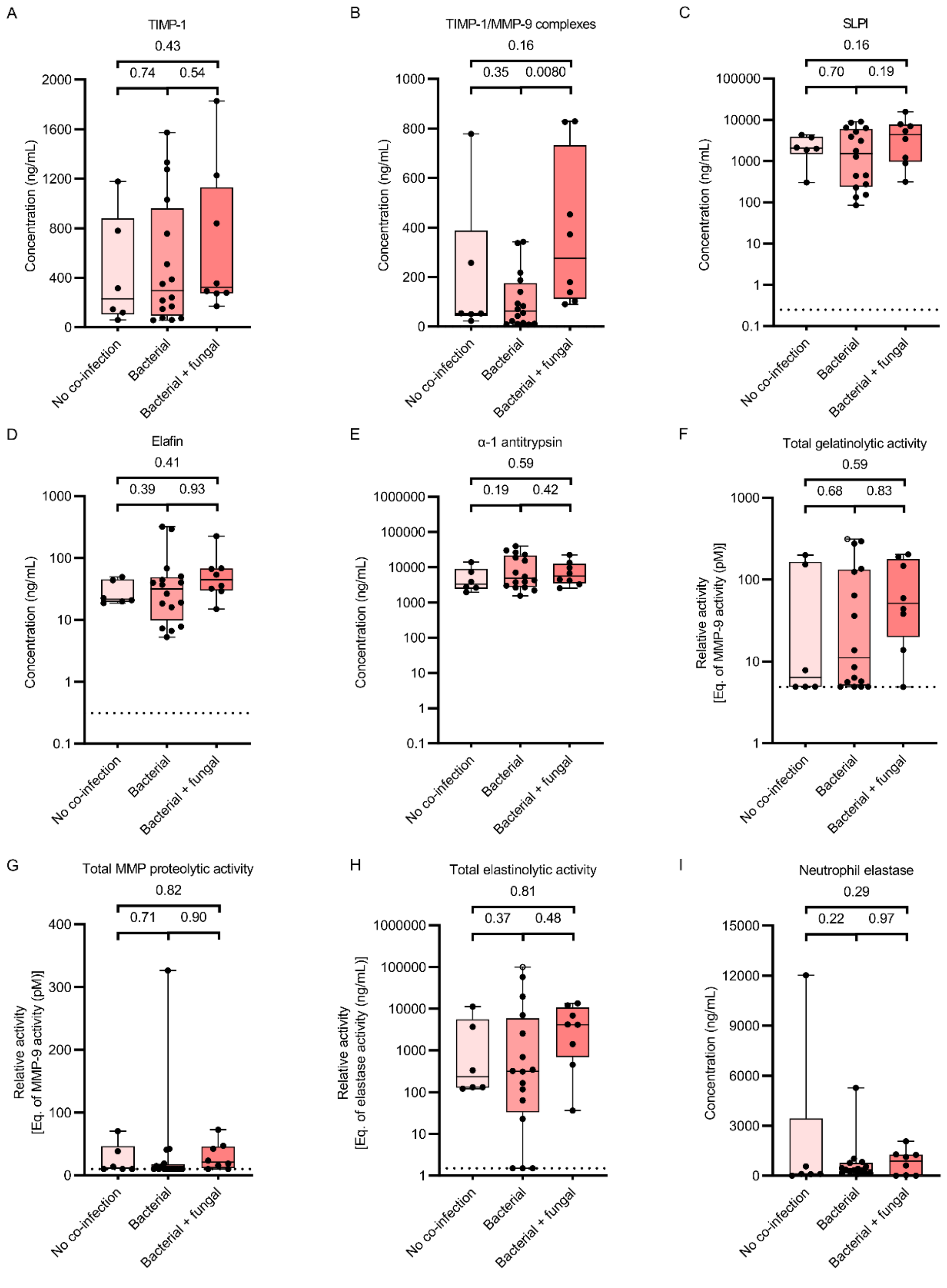

Supplemental Figure 5. Quantification of protease activity, protease and protease inhibitor levels in BAL fluid from patients with severe COVID-19, stratified by the presence and type of a co-infection. ELISA was used to determine concentrations of **(A)** TIMP-1, **(B)** TIMP-1/MMP-9 complexes, **(C)** SLPI, **(D)** elafin and **(E)** α-1 antitrypsin in BAL fluid samples from COVID-19 patients having no co-infection (n = 6) or having a bacterial (n = 16) or combined bacterial-fungal co-infection (n = 8). **(F)** Total gelatinolytic activity, as determined in a kinetic assay measuring degradation of a fluorogenic gelatin substrate, **(G)** total MMP proteolytic activity, as determined measuring degradation of a fluorogenic omni MMP substrate, **(H)** total elastinolytic activity, as determined measuring degradation of a fluorogenic elastin substrate and **(I)** neutrophil elastase levels (quantified by ELISA) were measured within the BAL fluid samples. Data are shown as box-and-whisker plots (box: median with interquartile range, whiskers: full data distribution) with each dot representing an individual patient sample. The dashed lines indicate the lower detection limits. Open symbols indicate values above the upper detection limit. Data were statistically analyzed by a linear mixed model with correction for multiple samples per patient using a random intercept model.

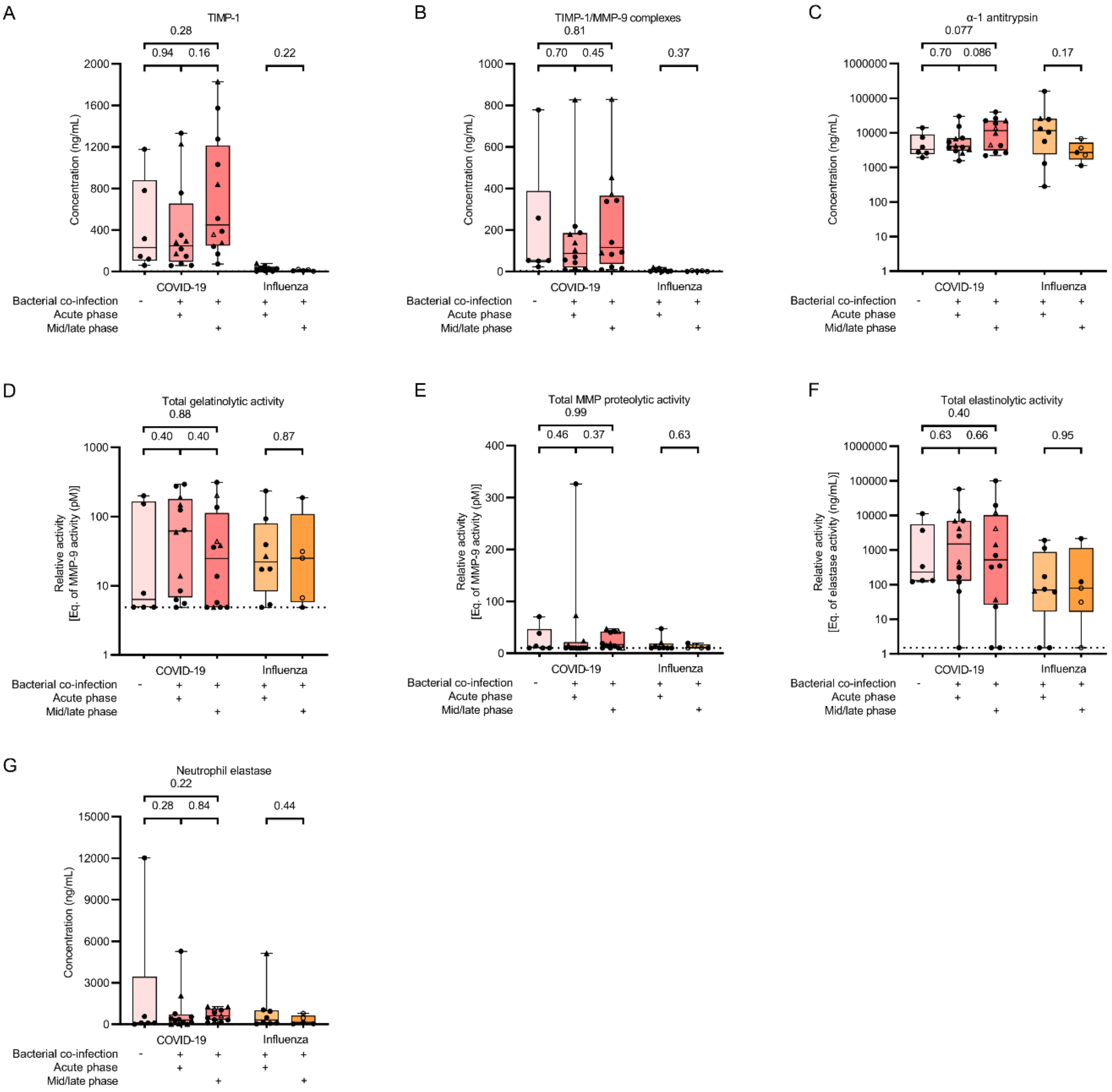

Supplemental Figure 6. Quantification of protease activity, protease and protease inhibitor levels in BAL fluid from patients with severe COVID-19 or influenza, stratified by the timing of a bacterial co-infection. ELISA was used to determine concentrations of **(A)** TIMP-1, **(B)** TIMP-1/MMP-9 complexes and **(C)** α-1 antitrypsin in BAL fluid samples from COVID-19 and influenza patients. COVID-19 samples were categorized based on the absence of a co-infection (n = 6) or the acute phase (n = 12) or mid/late phase of a bacterial co-infection (n = 12) based on the timing of the BAL sampling relative to the co-infection time course. Influenza patient samples were also categorized in the acute phase (n = 8) and the mid/late phase (n = 5) of a bacterial co-infection. **(D)** Total gelatinolytic activity, as determined in a kinetic assay measuring degradation of a fluorogenic gelatin substrate, **(E)** total MMP proteolytic activity, as determined measuring degradation of a fluorogenic omni MMP substrate, **(F)** total elastinolytic activity, as determined measuring degradation of a fluorogenic elastin substrate and **(G)** neutrophil elastase levels (quantified by ELISA) were measured within the BAL fluid samples. Data are shown as box-and-whisker plots (box: median with interquartile range, whiskers: full data distribution) with each dot representing an individual patient sample. The dashed lines indicate the lower detection limits. In the mid/late groups, open symbols indicate samples taken during the late phase of the bacterial co-infection, the others are taken during the midphase of the bacterial co-infection. Triangles indicate samples with an additional fungal co-infection. Data were statistically analyzed by a linear mixed model with correction for multiple samples per patient using a random intercept model or a Mann-Whitney test, where appropriate.

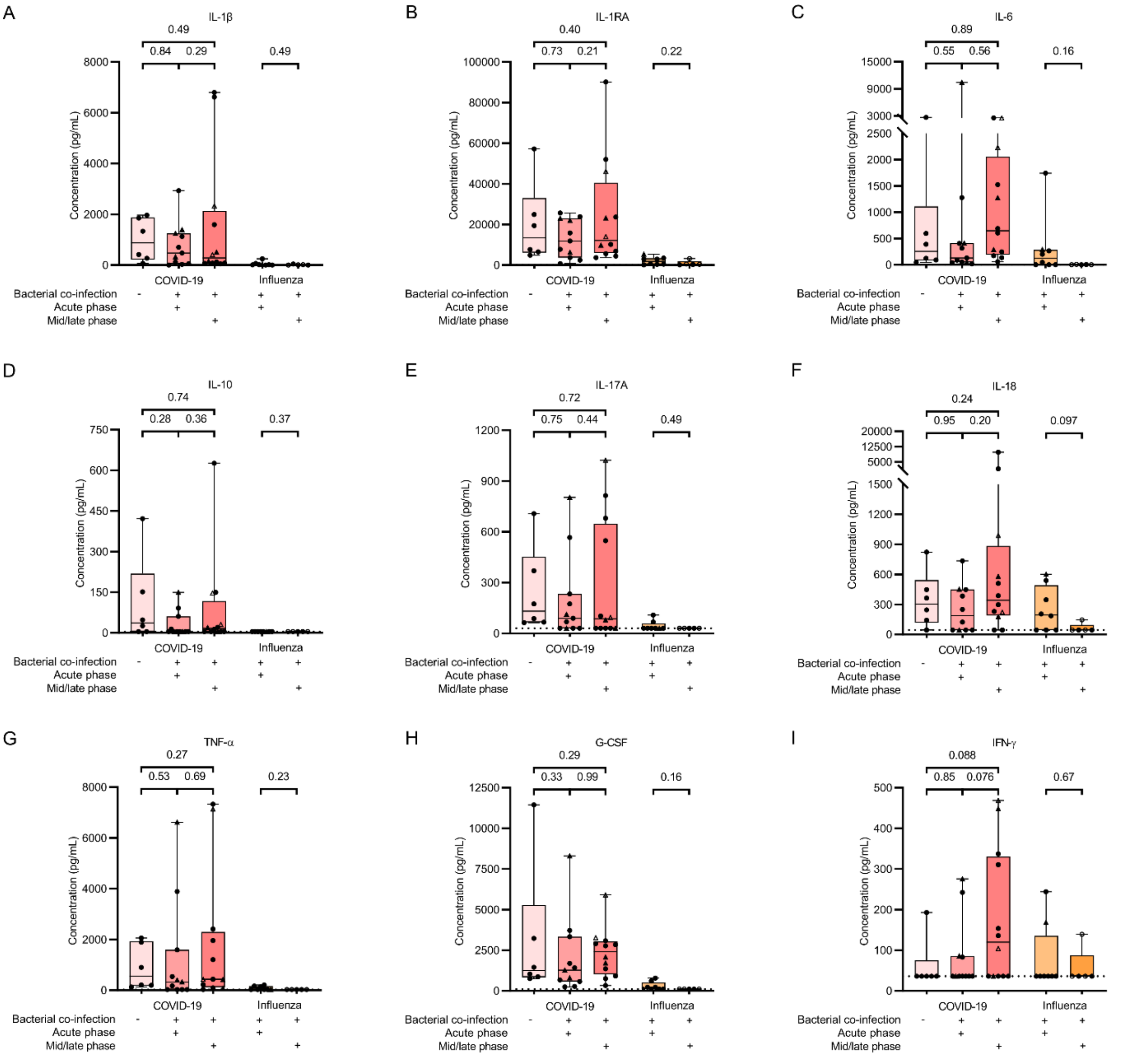

Supplemental Figure 7. Quantification of cytokine levels in BAL fluid from patients with severe COVID-19 or influenza, stratified by the timing of a bacterial co-infection. Multiplex technology was used to determine concentrations of **(A)** IL-1β, **(B)** IL-1RA, **(C)** IL-6, **(D)** IL-10, **(E)** IL-17A, **(F)** IL-18, **(G)** TNF-α, **(H)** G-CSF and **(I)** IFN-γ in BAL fluid samples from COVID-19 and influenza patients. COVID-19 samples were categorized based on the absence of a co-infection (n = 6) or the acute phase (n = 11) or mid/late phase of a bacterial co-infection (n = 12) based on the timing of the BAL sampling relative to the co-infection time course. Influenza patient samples were also categorized in the acute phase (n = 8) and the mid/late phase (n = 5) of a bacterial co-infection. Data are shown as box-and-whisker plots (box: median with interquartile range, whiskers: full data distribution) with each dot representing an individual patient sample. The dashed lines indicate the lower detection limits. In the mid/late groups, open symbols indicate samples taken during the late phase of the bacterial co-infection, the others are taken during the midphase of the bacterial co-infection. Triangles indicate samples with an additional fungal co-infection. Data were statistically analyzed by a linear mixed model with correction for multiple samples per patient using a random intercept model or a Mann-Whitney test, where appropriate.

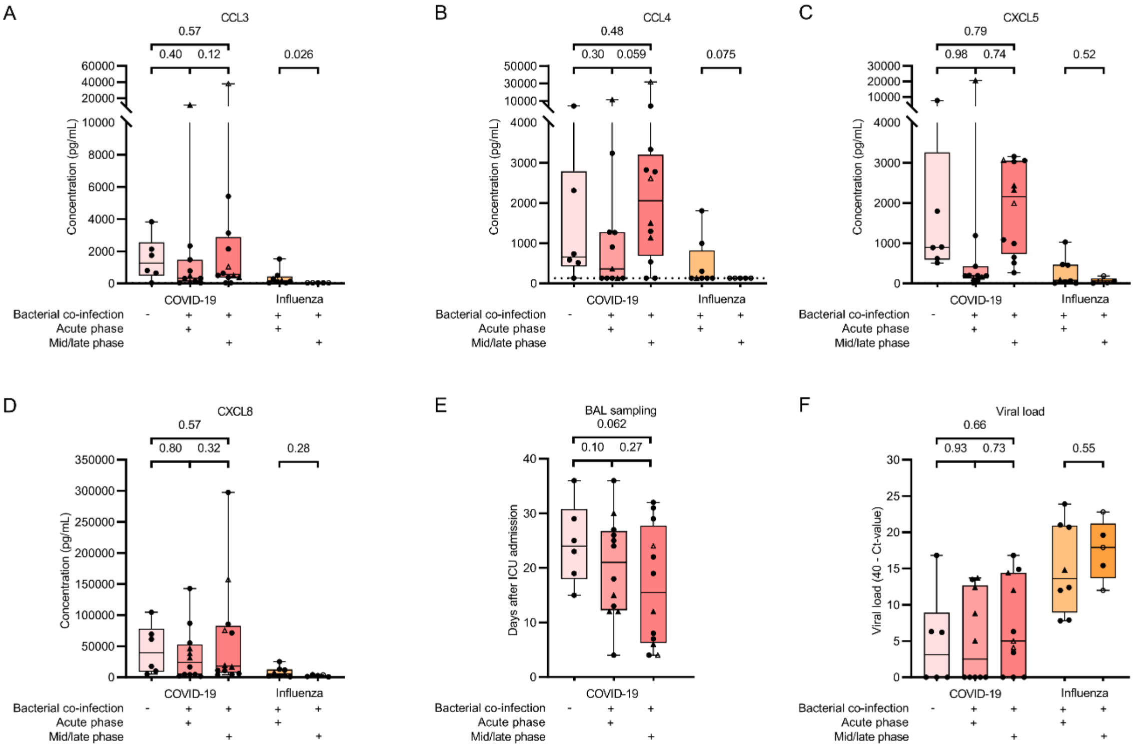

Supplemental Figure 8. Quantification of chemokine levels, timing of BAL sampling and determination of viral load in BAL fluid from patients with severe COVID-19 or influenza, stratified by the timing of a bacterial co-infection. Multiplex and ELISA technology was used to determine concentrations of **(A)** CCL3, **(B)** CCL4, **(C)** CXCL5 and **(D)** CXCL8 in BAL fluid samples from COVID-19 and influenza patients. (**E**) Timing of BAL fluid collection during ICU stay. (**F**) Determination of viral load in the BAL samples by RT-qPCR and described as 40- Ct-value with a value of zero representing no detection of viral RNA. COVID-19 samples were categorized based on the absence of a co-infection (*n* = 6) or the acute phase (*n* = 11) or mid/late phase of a bacterial co-infection (*n* = 12) based on the timing of the BAL sampling relative to the co-infection time course. Influenza patient samples were also categorized in the acute phase (*n* = 8) and the mid/late phase (*n* = 5) of a bacterial co-infection. Data are shown as box-and-whisker plots (box: median with interquartile range, whiskers: full data distribution) with each dot representing an individual patient sample. The dashed lines indicate the lower detection limits. In the mid/late groups, open symbols indicate samples taken during the late phase of the bacterial co-infection, the others are taken during the midphase of the bacterial co-infection. Triangles indicate samples with an additional fungal co-infection. Data were statistically analyzed by a linear mixed model with correction for multiple samples per patient using a random intercept model or a Mann-Whitney test, where appropriate.

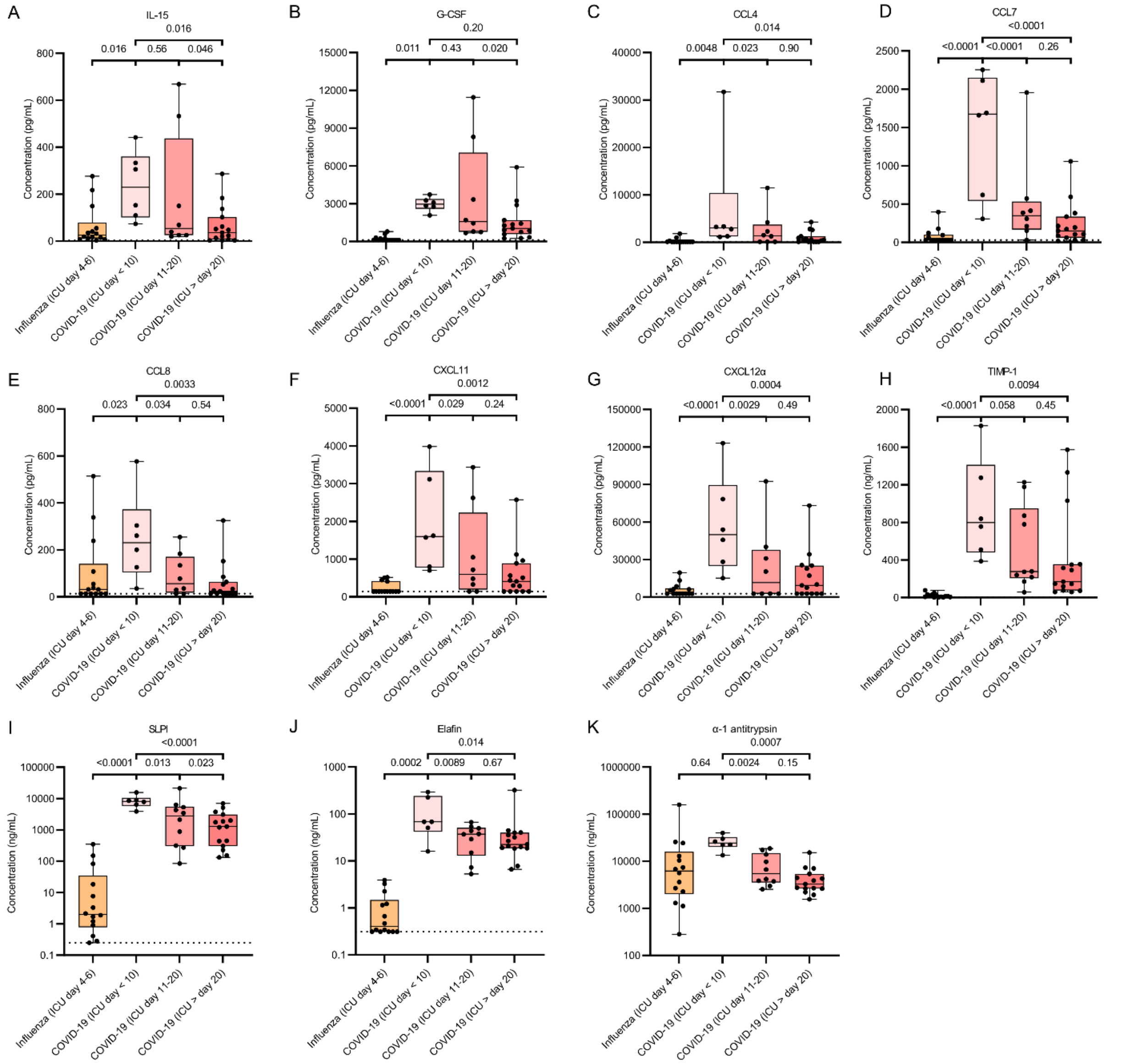

Supplemental Figure 9. Quantification of cytokine, chemokine and protease inhibitor levels in BAL fluid from patients with severe COVID-19 or influenza, stratified by the timing of BAL fluid collection during ICU stay. Multiplex and ELISA technology was used to determine concentrations of **(A)** IL-15, **(B)** G-CSF, **(C)** CCL4, **(D)** CCL7, (**E**) CCL8, (**F**) CXCL11, (**G**) CXCL12α, (**H**) TIMP-1, (**I**) SLPI, (**J**) elafin and (**K**) α-1 antitrypsin in BAL fluid samples from COVID-19 and influenza patients. COVID-19 samples were categorized according to the timing of BAL fluid collection since ICU admission in three groups: (1) <10 days after ICU admission (n = 6); (2) 10-20 days after ICU admission (n = 10) and (3) >20 days after ICU admission (n = 15). All influenza BAL samples were collected 4-6 days after ICU admission. Data are shown as box-and-whisker plots (box: median with interquartile range, whiskers: full data distribution) with each dot representing an individual patient sample. The dashed lines indicate the lower detection limits. Data were statistically analyzed by a linear mixed model with correction for multiple samples per patient using a random intercept model.

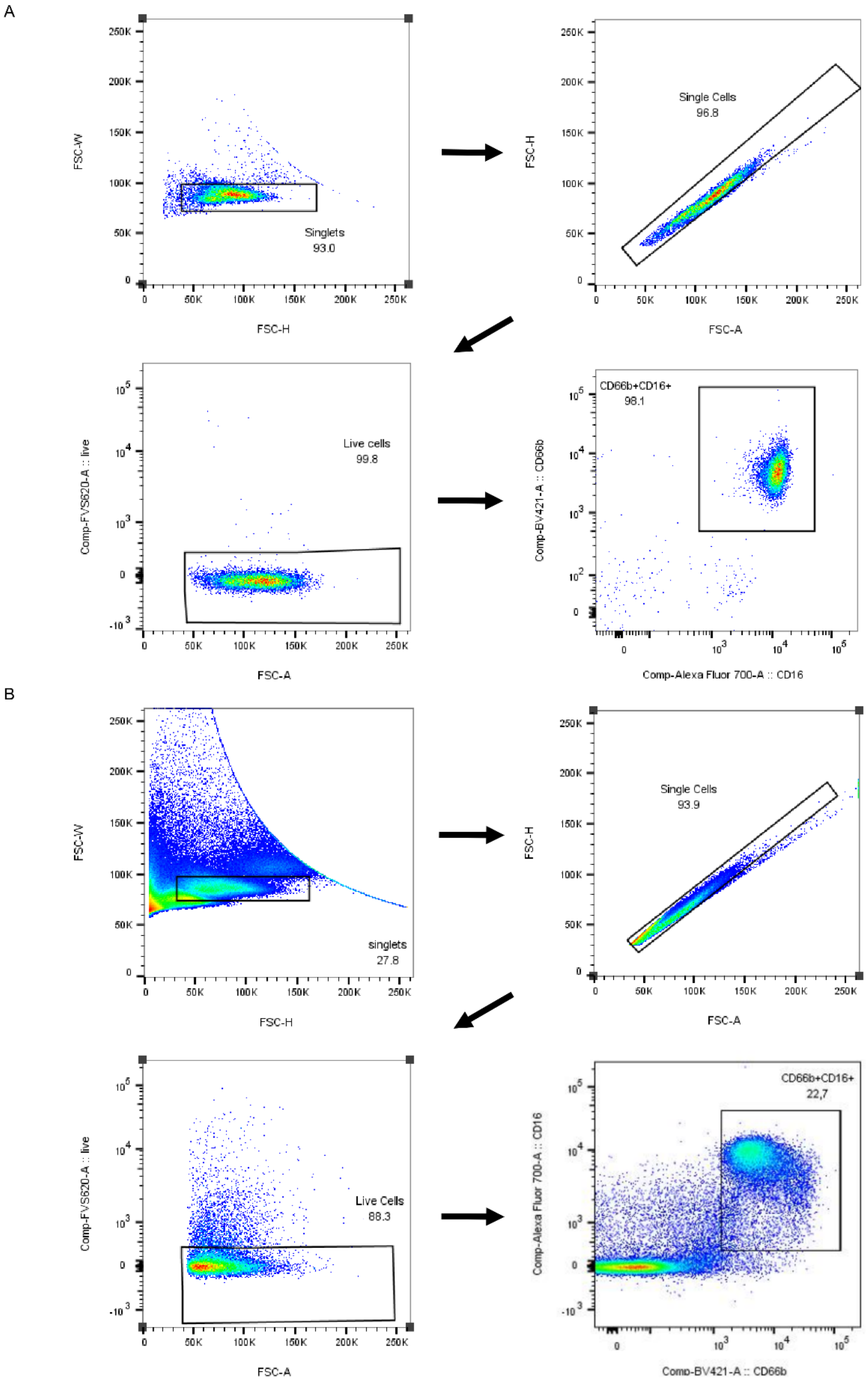

Supplemental Figure 10. Gating strategies for flow cytometry experiments. Gating strategy for flow cytometry analysis of **(A)** purified peripheral blood neutrophils and **(B)** BAL cells from patients with COVID-19. Single cells were gated based on forward scatter height, width and area (FSC-H, FSC-W, FSC-A). Dead cells were excluded by uptake of Fixable Viability Stain 620 (FVS620) or Zombie Aqua 516. Living, single cells were determined to be neutrophils if they expressed both CD16 and CD66b ($CD16^{+}CD66b^{+}$ cells).

Human antigen	Clone	Label	Host species	Company
CD10	HI10a	BV786	Mouse	BD Biosciences
CD11b	ICRF44	BV510	Mouse	BD Biosciences
CD11b	ICRF44	APC-Cy7	Mouse	Biolegend
CD11c	3.9	eFluor710	Mouse	eBioscience
CD14	61D3	PE-Cy7	Mouse	eBioscience
CD15	HI98	BUV395	Mouse	BD Biosciences
CD15	W6D3	BV786	Mouse	BD Biosciences
CD16	3G8	Alexa Fluor 700	Mouse	BD Biosciences
CD16	3G8	BUV395	Mouse	BD Biosciences
CD32	FLI8.26	BV711	Mouse	BD Biosciences
CD35	E11	FITC	Mouse	Biolegend
CD49d	9F10	BV711	Mouse	Biolegend
CD54	HA58	BV711	Mouse	BD Biosciences
CD62L	DREG56	APC	Mouse	eBioscience
CD63	H5C6	BV510	Mouse	BD Biosciences
CD64	10.1	eFluor710	Mouse	Invitrogen
CD66b	G10F5	BV421	Mouse	BD Biosciences
CD66b	G10F5	PerCP-Cy5.5	Mouse	Biolegend
CD69	H1.2F3	APC	Mouse	eBioscience
CD88 (C5aR)	S5/1	PerCP-Cy5.5	Mouse	Biolegend
CD181 (CXCR1)	5A12	PE	Mouse	BD Biosciences
CD182 (CXCR2)	6C6	FITC	Mouse	BD Biosciences
CD183 (CXCR3)	1C/CXCR3	BUV395	Mouse	BD Biosciences
CD184 (CXCR4)	12G5	BUV395	Mouse	BD Biosciences
CD191 (CCR1)	53504	Alexa Fluor 647	Mouse	BD Biosciences
CD192 (CCR2)	K036C2	Alexa Fluor 488	Mouse	Biolegend
CD282 (TLR2)	11G7	BV521	Mouse	BD Biosciences
CD284 (TLR4)	HTA125	FITC	Mouse	Invitrogen
CD286 (TLR6)	TLR6.127	PE	Mouse	Biolegend
CD289 (TLR9)	eB72-1665	APC	Mouse	eBioscience
BLTR1	203/14F11	BV510	Mouse	BD Biosciences
FPR1	5F1	Alexa Fluor 647	Mouse	BD Biosciences
FPR2	GM1D6	PE	Mouse	Santa Cruz
HLA-DQ	Tu169	Alexa Fluor 647	Mouse	BD Biosciences
HLA-DR	L243	BV650	Mouse	Biolegend
IL-1R1	FAB269P	PE	Mouse	R&D Systems
IL-1R2	REA744	FITC	Mouse	Miltenyi Biotec

Supplemental Table 1. Overview of antibodies used for flow cytometry. APC, allophycocyanin; BUV, Brilliant Ultraviolet; BV, Brilliant Violet; FITC, fluorescein isothiocyanate; PE, phycoerythrin; PerCP, Peridinin Chlorophyll Protein Complex.

SUPPLEMENTAL ACKNOWLEDGMENTS

CONTAGIOUS Consortium members & affiliations

Toine Mercier[5], Carine Wouters[6], Jan Gunst[8], Natalie Lorent[10], Pierre Van Mol[11], Diether Lambrechts[11], Yannick Van Herck[12], Francesca Maria Bosisio[13], Thomas Tousseyn[13], Birgit Weynand[13], Frederik De Smet[14], Abhishek Garg[15], Stephanie Humblet-Baron[16], Kim Martinod[17], Sabine Tejpar[18], Karin Thevissen[19]

Affiliations:

[5]Laboratory of Clinical Bacteriology and Mycology, Department of Microbiology, Immunology and Transplantation, KU Leuven; Leuven, Belgium.

[6]Laboratory of Immunobiology, Department of Microbiology, Immunology and Transplantation, Rega Institute, KU Leuven; Leuven, Belgium.

[8]Laboratory of Intensive Care Medicine, Department of Cellular and Molecular Medicine, KU Leuven; Leuven, Belgium.

[10]Department of Pneumology, University Hospitals Leuven; Leuven, Belgium.

[11]Laboratory of Translational Genetics, Department of Human Genetics, VIB-KU Leuven; Leuven, Belgium.

[12]Laboratory of Experimental Oncology, Department of Oncology, KU Leuven; Leuven, Belgium.

[13]Translational Cell & Tissue Research, Department of Imaging & Pathology, KU Leuven; Leuven, Belgium.

[14]Laboratory for Precision Cancer Medicine, Translational Cell and Tissue Research, Department of Imaging & Pathology, KU Leuven; Leuven, Belgium.

[15]Laboratory for Cell Stress & Immunity (CSI), Department of Cellular and Molecular Medicine (CMM), KU Leuven; Leuven, Belgium.

[16]Adaptive Immunology, Department of Microbiology, Immunology and Transplantation, KU Leuven; Leuven, Belgium.

[17]Centre for Molecular and Vascular Biology, Department of Cardiovascular Sciences, KU Leuven; Leuven, Belgium.

[18]Molecular Digestive Oncology, Department of Oncology, KU Leuven; Leuven, Belgium.

[19]Centre of Microbial and Plant Genetics, Department of Microbial and Molecular Systems (MS), KU Leuven; Leuven, Belgium.
